# Moringa Oleifera Lam. in Cardiometabolic Disorders: A Systematic Review of Recent Studies and Possible Mechanism of Actions

**DOI:** 10.3389/fphar.2022.792794

**Published:** 2022-03-30

**Authors:** Melva Louisa, Cyntia Gracesella Hutami Patintingan, Bantari W. K. Wardhani

**Affiliations:** ^1^ Department of Pharmacology and Therapeutics, Faculty of Medicine, Universitas Indonesia, Jakarta, Indonesia; ^2^ Master Program in Biomedical Sciences, Faculty of Medicine, Universitas Indonesia, Jakarta, Indonesia; ^3^ Department of Pharmacology, Faculty of Military Pharmacy, Indonesia Defense University, West Java, Indonesia

**Keywords:** metabolic syndrome, antioxidant, antiinflammation, flavonoids, lectin, Moringa oleifera (Moringaceae)

## Abstract

Cardiometabolic disorders (CMD) have become a global emergency and increasing burden on health and economic problems. Due to the increasing need for new drugs for cardiometabolic diseases, many alternative medicines from plants have been considered and studied. *Moringa oleifera* Lam. (MO), one of the native plants from several Asian countries, has been used empirically by people for various kinds of illnesses. In the present systematic review, we aimed to investigate the recent studies of MO in CMD and its possible mechanism of action. We systematically searched from three databases and summarized the data. This review includes a total of 108 papers in nonclinical studies and clinical trials of MO in cardiometabolic-related disorders. *Moringa oleifera*, extracts or isolated compound, exerts its effect on CMD through its antioxidative, anti-inflammatory actions resulting in the modulation in glucose and lipid metabolism and the preservation of target organ damage. Several studies supported the beneficial effect of MO in regulating the gut microbiome, which generates the diversity of gut microbiota and reduces the number of harmful bacteria in the caecum. Molecular actions that have been studied include the suppression of NF-kB translocation, upregulation of the Nrf2/Keap1 pathway, stimulation of total antioxidant capacity by reducing PKCζ activation, and inhibiting the Nox4 protein expression and several other proposed mechanisms. The present review found substantial evidence supporting the potential benefits of *Moringa oleifera* in cardiovascular or metabolic disorders.

## 1 Introduction

In recent years, non-communicable diseases, specifically cardiometabolic disorders (CMD), have been steadily rising. The CMD is a collection of metabolic abnormalities primarily defined by insulin resistance, type 2 diabetes mellitus, dyslipidemia, hypertension, and central obesity (Akhigbe and Ajayi, 2021). Over the last decades, the surge in number occurred rapidly in high-income nations and in low- and middle-income countries (LMICs) (Miranda et al., 2019; Maffetone and Laursen, 2020). The recent disruption of COVID-19 pandemics made patients’ cardiovascular illnesses become the most common comorbidities associated with increased mortality and morbidity (Ganatra et al., 2020; Kulkarni et al., 2020). CMD caused a massive burden on socioeconomic aspects throughout the globe (Miranda et al., 2019). Up to date, researchers are still searching for new and effective therapeutic options. Several alternative treatments, including phytotherapy, are being investigated for future clinical use (Nieves and Baum, 2017). *Moringa oleifera* Lam. (MO) is one of the natural-derived medicines being studied for the treatment of CMD.

*MO* is an indigenous plant from several parts of Asia and has been used empirically by people for nutritional and health benefits. Traditional use of MO includes the treatment of fevers, diarrhea, antibacterial, tumor, inflammation, toothache, cardiac stimulants, and boosting the immune system (Abd Rani et al., 2018; Dhakad et al., 2019; Islam et al., 2021). Studies have been conducted using various kinds of MO extracts from seeds, oils, leaves, roots, or flowers to study their biological effects (Anudeep et al., 2016; Abd Rani et al., 2018; Huang et al., 2020; Islam et al., 2021). Bioactive compounds of MO were reported from numerous studies, including flavonoids, phenolic acids, terpenes, alkaloids, sterols, glucosinolate, and isothiocyanate (Stohs and Hartman, 2015; Jaja-Chimedza et al., 2017; Abd Rani et al., 2018; Liao et al., 2018; Dhakad et al., 2019; Fahey et al., 2019; Huang et al., 2020). Moreover, MO has been described as demonstrating a rich nutritional profile with high amounts of amino acids, fatty acids, polysaccharides, vitamins, and minerals (Anudeep et al., 2016; Dhakad et al., 2019; Li C. et al., 2020; Islam et al., 2021). The bioactive and nutritional contents of MO contribute to its potent antioxidant, anti-inflammatory, antibacterial, and immunomodulatory activities in many health-related problems (Stohs and Hartman, 2015; Abd Rani et al., 2018; Dhakad et al., 2019; Islam et al., 2021).

The benefit of MO for the use in CMD-related diseases such as diabetes, hypertension, dyslipidemia, and related complications has been studied in numerous individual studies (Ndong et al., 2007; Ouédraogo et al., 2013; Aa et al., 2017; Abarikwu et al., 2017; Paula et al., 2017; Wang et al., 2017; Aju et al., 2019; Bao et al., 2020; Abd-Elhakim et al., 2021; Abu-Zeid et al., 2021). In the present systematic review, we conducted a thorough database search and compiled studies on the effect and possible mechanism of *MO* actions for CMD treatment. Preclinical data, as well as the available clinical data in humans, are appraised and discussed.

## 2 Methods

### 2.1 Search Strategy

We searched several electronic databases: PubMed, Scopus, Google Scholar, and Cochrane Central Register of Controlled Trials from inception to 1st of August 2021. MeSH and keywords were used in the search strategy (Supplementary Table S1). No time and language restrictions were applied. The bibliography of all relevant manuscripts was checked to find potential missing publications.

Studies appraised were *in vitro*/*in vivo*/human studies of MO in cardiometabolic disorders, including, but not limited to, diabetes mellitus, obesity, hyperglycemia, dyslipidemia, or hypertension. We included randomized or non-randomized controlled trials and experimental *in vivo* or *in vitro* studies. Articles with irrelevant study designs such as *in silico*, study protocols, reviews, surveys, or observational studies were excluded. Papers that used MO with other agents in non-mammalian species and conditions other than CMD were also excluded.

### 2.2 Data Collection

Two reviewers independently extracted data from studies meeting eligibility criteria (BW and CG). Extracted data were subsequently checked by a third reviewer (ML). Discussions by the three reviewers resolved discrepancies. The following nonclinical data were retrieved from each study: first author, year of publication, MO extracts used, type of experimental models, primary findings, and mechanism of actions. We reported the Authors and year of publication, MO extracts used, sample size, the dose of MO and type of control, duration, and effects for clinical trials included. Safety results were reported in nonclinical and clinical studies when available.

## 3 Results

### 3.1 Study Selection

**FIGURE 1:**
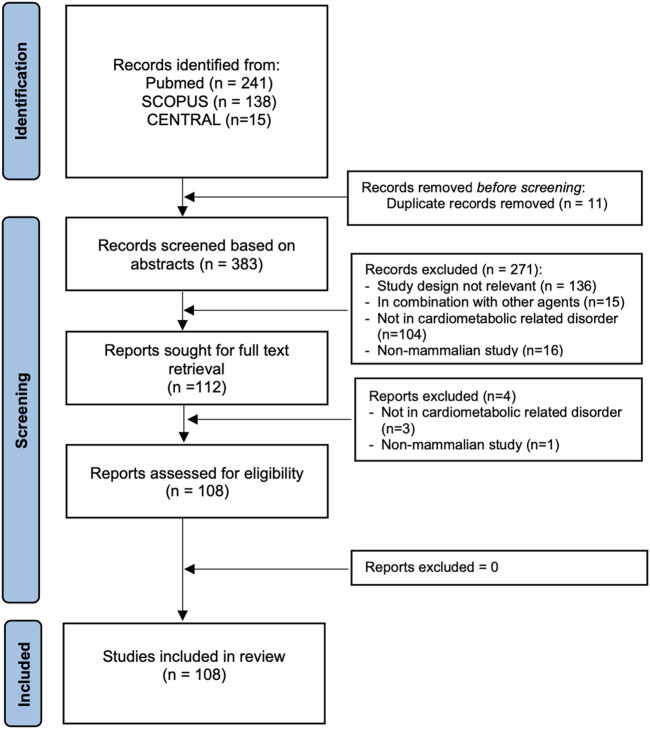
Flow of article selection in the study.

The search resulted in 108 articles from the three databases. The process of study selection is provided in Figure 1. We summarize the effects of *MO* in cardiometabolic-related disorders from a total of 108 articles: 102 reported nonclinical studies (Supplementary Table S2) and seven in clinical trials (Supplementary Table S3). Ezzat et al. (2020) wrote both nonclinical and clinical studies in one manuscript.

Most studies used extracts rather than isolated compounds from MO. Next, the solvents used were aqueous, ethanol, methanol, ethyl acetate fractions, and petroleum ether. Aqueous or hydro alcohol-based extraction resulted in the highest yield of the preparation. Although most of the manuscripts reported the method of extractions or purification, only 40 out of the 108 manuscripts reported the exact yield of the preparation from the raw material (Supplementary Table S2 and Supplementary Table S3).

Several isolated compounds were used in the study: isothiocyanates (Waterman et al., 2015; Huang et al., 2020), niazirin (Wang et al., 2019a; Bao et al., 2020), β-sitosterol (Liao et al., 2018). N, α, L-rhamnopyranosyl vincosamide, (Panda et al., 2013; Cheraghi et al., 2017), 1-O-(4-hydroxymethylphenyl)-α-L-rhamnopyranoside (MPG) (Sun et al., 2019). Additionally, some studies utilized isolated polysaccharides, proteins, and lectins from MO, which include the following: polysaccharides MRP-1 (Cui et al., 2019), novel polysaccharides from MO leaves (Li C. et al., 2020), water-soluble lectins (Araújo et al., 2013; Yurre et al., 2020; Vera-Nunez et al., 2021), coagulant MO lectin (Araújo et al., 2013), isolated protein from MO leaves (Paula et al., 2017).

### 3.2 Effects and Mechanism of MO Actions From Nonclinical Studies

#### 3.2.1 Antioxidative Effects

Increased oxidative stress has long been identified as the common etiology in cardiovascular diseases. Excessive reactive oxygen species (ROS) activate signaling pathways resulting in epigenetic dysregulation, chronic inflammation, endothelial dysfunction, and ultimately in apoptotic cell death (Santilli et al., 2015; Senoner and Dichtl, 2019).

We found robust and consistent data on the MO antioxidative effects in CMD-related animal models. Fifty-two studies provided the antioxidative mechanism of MO concerning its anti-hyperglycemia, anti-hyperlipidemia, antihypertensive effects, and protective effects in the vascular, kidney, and cardiac. MO lowers blood glucose levels and improves insulin secretion in a diabetic rat model by reducing MDA and 8-OH-dG, increasing GSH concentrations, GPx, SOD, and catalase activities (Gupta et al., 2012; Al-Malki and El Rabey, 2015; Arise et al., 2016; Abd Eldaim et al., 2017; Omodanisi et al., 2017; Paula et al., 2017; Tang et al., 2017; Umar et al., 2018; Aju et al., 2019; Abdel-Daim et al., 2020; Oldoni et al., 2021).

Physiologically, the human body exhibits important internal regulators as an antioxidant defense in reactive oxidants generated in the body from various exposures. The nuclear factor erythroid 2-related factor (Nrf2) is one of the regulators of cellular response against oxidative stress and electrophiles (Ma, 2013). Nrf2, a transcription factor, acts by regulating the expression of genes containing antioxidant-responsive elements (ARE). Nrf2 is controlled by repressor protein Keap1 that serves as a molecular sensor for changes in cellular redox balances (Tkachev et al., 2011). Numerous studies demonstrated the dysregulation of Nrf2/Keap1/ARE pathways in many chronic diseases, including diabetes mellitus, obesity, and atherosclerosis (Cuadrado et al., 2019; da Costa et al., 2019).

MO leaf or seed extracts provided upregulation of Nrf2 expressions and downstream Nrf2 pathways: HO-1, NQO1, and GSTP1 in multiple rodent models. Upregulation of Nrf2 signaling was followed by increased antioxidant markers (MDA, SOD, GSH) and prevention of further organ damage (Jaja-Chimedza et al., 2017; Abdou et al., 2019; Soliman et al., 2020).

Another pathway associated with ROS production is PKC/Nox4 signaling pathway. PKC/Nox4 overactivation has been linked with excessive ROS production and relates to the development of metabolic diseases (Thallas-Bonke et al., 2014). Niazirin, a novel phenolic glycoside isolated from aqueous MO seed, was found to increase total antioxidant capacity by reducing high-glucose-induced PKCζ activation and inhibiting the Nox4 protein expression (Wang et al., 2019a).

Reports indicate that high concentrations of flavonoids and phenolic acids in MO are the main contributors to MO’s high level of antioxidative action (Chumark et al., 2008; Aa et al., 2017; Abd Eldaim et al., 2017; Paula et al., 2017; Aju et al., 2019). Rutin, quercetin, kaempferol, isoquercetin, isorhamnetin, apigenin, and luteolin are among the most prevalent flavonoids found in MO (Abd Rani et al., 2018). In addition, the study by Paula et al. (2017) reported that protein isolate from MO leaves exert potent antioxidant activities supporting its antidiabetic activity in alloxan-induced diabetes in mice.

#### 3.2.2 Antiinflammation

An increasing amount of studies demonstrate that inflammation has been linked to the progress of CMD. Researchers showed that targeting inflammation in animal models reduces cardiac and vascular damage, slows the development of illness, and stimulates the healing process (Ruparelia et al., 2017). In cardiovascular diseases, the production of various inflammatory cytokines, including TNF-α, IL-1β, and IL-6, leads to tissue damage, promoting atherosclerotic plaque formation, reduced vascular function, and progression of heart failure (Amin et al., 2020). MO extracts have been studied for their anti-inflammatory properties to antidiabetic, antihyperlipidemic, and organ damage protection effects.

*In vitro* studies using RAW246.7, murine macrophage cells, mice macrophage cells, and human MDM revealed the anti-inflammatory mechanism of MO. MO acts by inhibiting translocation of NF-κB (p65) translocation into the nucleus, resulting in the reduction of p65, phospho-IκB-α, and COX-2 proteins levels (Sinha et al., 2011; Luetragoon et al., 2020). Additionally, IL-10 and IκB-α were upregulated in a dose-dependent manner after MO flower hydroethanolic extracts were administered in macrophage cells stimulated with lipopolysaccharides (Tan et al., 2015). Following treatment with MO in a diabetes rat model or chemically-induced liver and kidney damage, MO was found to provide its therapeutic effect by reducing the expressions of several pro-inflammatory cytokines TNFα, IL-1β, IL-6, and IL-12 (Joung et al., 2017; Azevedo Í et al., 2018; Abdou et al., 2019; Chin et al., 2019; Abdel Fattah et al., 2020; Abd-Elhakim et al., 2021; Abou-Zeid et al., 2021).

In addition to cytokines, monocyte chemoattractant protein (MCP-1) is one of the critical chemokines in macrophage recruitment (Deshmane et al., 2009; Cranford et al., 2016). In a study using FVB/N strain, Cranford et al. (2016) demonstrated that MCP-1 is essential for adipose tissue protection following a high-fat diet.

An active compound from MO seeds, 1-O-(4-hydroxymethylphenyl)-α-L-rhamnopyranoside (MPG) was used to treat CCl4 liver injury in ICR mice. MO caused a reduction in TNF-α, IL-1β, and MCP-1 levels in the liver and elevated IL-10 levels in the systemic circulation (Sun et al., 2019). Another study utilizing MO leaves as a film dressing in streptozotocin, and high-fat-induced rats resulted in a faster wound healing rate, followed by downregulation of pro-inflammatory cytokines and MCP-1 reduction (Chin et al., 2019). However, an enhancement of inflammatory response occurred, marked by upregulations of TNF-α, IL-1β, IL-6, and MCP-1 in the colon when MO leaves aqueous extracts were given to healthy C57BL/6-J mice (Gao et al., 2017).

Studies suggested the relationship between inflammation in the colon to CMD (Bunu et al., 2019; Verdugo-Meza et al., 2020). Several studies showed that high-fat diet-induced obesity in animal models causes colitis by exacerbating mucosal oxidative stress, which quickly increases mucosal inflammation and permeability of the intestinal mucosal barrier (Bilski et al., 2019; Li et al., 2019; Li and Li, 2020). Furthermore, chronic inflammation in the colon disrupts the gut barrier, allowing microbes to infiltrate the submucosa and increase the likelihood of gut-derived metabolites being transported from the gut to the liver and pancreas. As a result, the chance of developing insulin resistance was likely enhanced in colitis (Bunu et al., 2019; Verdugo-Meza et al., 2020).

Nitric oxide (NO), a signaling messenger, engage with cytokines in providing regulatory control in the cardiovascular system. Endothelial dysfunction, hypertension, diabetes mellitus, thrombosis, and stroke, have all been related to dysregulation in NO bioavailability (Naseem, 2005; Napoli and Ignarro, 2009). MO treatment has been shown to suppress NO production and iNOS expression and reduce pro-inflammatory cytokines and MCP-1.

Liao et al., in their study, identified β-sitosterol isolated from MO woody stems as an anti-inflammatory agent. β-sitosterol was shown to decrease the secretion of inflammatory cytokines, IL-1β, IL-6, IL-8, and TNF-α in a study using HaCaT and macrophages. In the same study, NRLP3 expression, caspase-1 activation, and NF-KB production were also inhibited by β-sitosterol (Liao et al., 2018).

As a comparison, one contradictory result was demonstrated by Li et al. They studied a novel polysaccharide from MO leaves in RAW246.7, namely MOP-3, which resulted in an enhancement of pinocytosis, ROS, NO, IL-6, and TNF-α at the range of 31.3–500 μg/ml (Li C. et al., 2020).

#### 3.2.3 Glucose and Lipid Metabolism

Antihyperglycemic activity of MO was among the most widely studied and has been applied in several clinical trials. Numerous bodies of evidence reported that MO afforded blood-glucose-lowering effect through its activity either in increasing insulin secretion (insulin secretagogue) (Tang et al., 2017) or improving insulin resistance (Wang et al., 2017; Umar et al., 2018; Bao et al., 2020; Vera-Nunez et al., 2021). Wang et al. (2017) reported that three isolated compounds extracted from the resin adsorption extract of MO seeds were shown to stimulate glucose consumption in insulin resistance cells and lower blood glucose levels in streptozotocin-induced rats. Less degenerative changes of beta cells were found in alloxan-induced diabetic rats treated with methanol extracts of MO root (Umar et al., 2018). Improvement of insulin resistance was also demonstrated by water purified extract of MO rich in lectins, as shown by the reduction of intraperitoneal glucose tolerance test (Umar et al., 2018). Bao et al. (2020) demonstrated that niazirin, a phenolic glycoside from MO, alleviates insulin resistance, as shown by the reduction of HOMA-IR, acting as an AMPK agonist, and decreasing hepatic metabolism in db/db mice. Yet, Yurre et al. (2020) reported that water-soluble lectins isolated from MO seeds did not improve insulin resistance.

Insulin signaling regulates glucose and lipid homeostasis, mainly in the liver, skeletal muscle, and adipose tissue. Several regulators of insulin signaling are the critical steps in modulating insulin response (Boucher et al., 2014). Insulin receptor substrate proteins (IRS1 and IRS2) are essential targets for insulin receptors for glucose metabolism. Downregulation of IRS was found in insulin-resistant animals and humans. Further, disruption in IRS1/2 phosphorylation by insulin-dependent kinase was found in hyperglycemia conditions (Copps and White, 2012). Waterman et al. reported that isothiocyanate-rich MO extracts reduce insulin resistance in mice by suppressing gluconeogenesis in the liver. Reduction in liver gluconeogenesis by MO resulted in insulin signaling enhancement. MO increases phosphorylation of IRS-1 in the liver and IRS-1 and IRS-2 and insulin receptor-β (IR-β) in the muscle. Moreover, glucose transporter 4 (GLUT4), which was an insulin-regulated transporter, was increased in the muscle after MO treatment (Waterman et al., 2015).

Further, MO leaves ethanol extract was shown to reduce glucose absorption by inhibiting α-amylase, enhancing gut motility, and decreasing starch catabolism (Al-Malki and El Rabey, 2015; Azad et al., 2017). However, in the study by Azad et al. (2017) MO did not affect insulin secretion. Additionally, Shaikh et al. (2020) reported *in vitro* activity of MO roots and seeds extracts in inhibiting pancreatic amylase activity.

Another regulator in carbohydrate metabolism is AMPK. AMPK resulted in many downstream targets and influenced cellular processes such as lipid metabolism (Shirwany and Zou, 2010). Bao et al. reported that niazirin from MO improved carbohydrate and lipid metabolism via the AMPK signaling pathway. In the study, they suggested that niazirin might be an AMPK agonist. The result showed that niazirin activated AMPK to regulate carbohydrate metabolism in the liver. As a result, the downstream of AMPK control gluconeogenesis, phosphoenolpyruvate carboxykinase (PEPCK) and Glucose-6-phosphatase, were further regulated (Bao et al., 2020). MO is also rich in quercetin, previously described as AMPK activators (Dhanya et al., 2017).

Activation of AMPK was also described to regulate many more transcription factors, including Peroxisome proliferator-activated receptor alpha (PPARα) and Sterol regulatory element-binding protein (SREBP-1) controls fatty acid metabolism (Shirwany and Zou, 2010). Peroxisome proliferator-activated receptors (PPARs) are nuclear receptors that regulate lipid and glucose metabolism. In metabolic syndromes, studies described three isoforms (α, δ, and γ). PPAR-alpha (PPAR-α) is a major transcriptional factor regulating free fatty acid oxidations and is ubiquitous in the liver, heart, and skeletal muscle. PPAR-δ acts as a regulator of fatty acid oxidation in tissues in which PPAR-α are less expressed, while activation of PPAR-γ stimulates triglyceride storage (Ferré, 2004).

Ezzat et al. reported that MO leaves ethanol extract increased the expression of PPAR-α, suppressed HMG-CoA reductase, fatty acid synthase, increasing adiponectin and GLUT4 levels in the adipose tissue of high-fat diet-induced obesity rat model. Collectively, the signaling pathway mediated by MO extracts reduced body weight, adiposity index, HOMA-IR, and cholesterol levels (Ezzat et al., 2020). Similarly, Reddy et al. (2017) demonstrate that MO leaves polyphenol suppressed HMG-CoA reductase activity, resulting in reducing plasma cholesterol levels. In contrast, in an *in vitro* study in HepG2 cells, methanol extract of MO was found to downregulate PPARα1 and PPARγ gene expression (Sangkitikomol et al., 2014).

Additionally, in a mice model of high fat-induced obesity, MO fermented extracts demonstrated its efficacy in reducing hepatic lipid accumulation by causing downregulation of lipogenic genes (ACC, FAS, C/ENPα, SREBP1c, LPL), lipid oxidative genes [CD36, ACOX1, CPT1 (NFM), HSL], and oxidative stress genes (UCP2 and UCP3). In addition, MO alleviates the expressions of ER relation genes (Joung et al., 2017).

Pyruvate carboxylase (PC) is essential in gluconeogenesis and adipogenesis. PC acts by providing oxaloacetate to convert phosphoenolpyruvate by phosphoenolpyruvate carboxykinase (PEPCK). While PC participates in the *de novo* fatty acid synthesis pathway, PC downregulation contributes to insulin resistance in an obesity-induced diabetic model (Jitrapakdee et al., 2008). MO leaves extract treatment in alloxan-induced diabetic rats and tilmicosin-induced cardiac injury caused by the increased PC activity (Abd El Latif et al., 2014; Abd Eldaim et al., 2017; Khalil et al., 2020). In comparison, a study in 3T3-L1 adipocytes showed that isothiocyanates from MO seeds inhibited intracellular lipid accumulation (Huang et al., 2020).

Recently, researchers have been targeting dipeptidyl-peptidase-4 (DPP-4) as a new target in diabetes mellitus. DPP4 is a regulatory protease with a unique role in activating intracellular signal transduction. One of the DPP4 substrates is glucagon-like-peptide (GLP-1), an intestinal hormone with a glucose-lowering effect. DPP4 activity was negatively correlated with GLP-1 activity. Thus, inhibition of DPP4 was sought by researchers to increase GLP-1 (Deacon, 2019; Deacon, 2020).

A study by Yang et al. (2020) demonstrated that the bioactive compound of MO: O-Ethyl-4-[(α-l-rhamnosyloxy) benzyl] carbamate exhibit DPP-IV inhibiting activity at 798 nM using *in vitro* screening method.

#### 3.2.4 Antihypertensive

Seven studies demonstrated the effect of MO in the hypertensive rat model. However, not all studies signify the role of MO in reducing blood pressure. Yet, all studies demonstrate the favorable effects of using MO in the hypertensive model. MO provides cardio- and vasculoprotective effects despite its blood pressure-lowering effect (Randriamboavonjy et al., 2016; Randriamboavonjy et al., 2017; Aekthammarat et al., 2020a; Aekthammarat et al., 2020b).

Randriamboavonjy et al. demonstrated that the treatment of MO seed powder at 750 mg/day for 8 weeks in spontaneously hypertensive rats reduced the thickness of the left ventricle’s anterior and posterior walls. Improvements followed the reduction in cardiac fibrosis. Additionally, MO was demonstrated to increase cardiac PPARα and PPARγ expressions, followed by a decrease in triglyceride plasma concentrations (Randriamboavonjy et al., 2016). Randriamboavonjy et al. confirmed their results by demonstrating that MO seed powder alleviates oxidative and nitrosative stress in animal models of hypertension. MO lowered free 8-isoprostane concentrations and p22^phox^ and p47^phox^ expressions while increasing the antioxidant enzyme superoxide dismutase activity. Simultaneously, MO decreases endothelial vascular tone in hypertensive rats (Randriamboavonjy et al., 2017).

Aekthammarat et al. studied the antihypertensive effect of MO aqueous leaves extracts in L-name-induced hypertension. In their study, Aekthammarat et al. proved that MO could reduce blood pressure and tachycardia by reducing hyperreactivity of adrenergic mediated contraction, oxidative stress (as shown by the decline in MDA, SOD, and CAT), and induced NO production (Aekthammarat et al., 2019; Aekthammarat et al., 2020a; Aekthammarat et al., 2020b). At the same time, in spontaneously hypertensive rats, MO aqueous leaves exhibit blood-pressure-lowering activity by inhibiting T-cell proliferation and anti-CD3-stimulated T-cell blastogenesis. Activated T cells resulted in tyrosine phosphorylation, LAT activation (linker for T cells), and PLCγ (phospholipase Cγ). Moreover, PLCγ activation leads to the rise in cytoplasm calcium concentration and smooth muscle contraction in the vascular system. The process caused an increase in peripheral vascular resistance and, thus, increased blood pressure. In this study, the inhibition of proliferation and anti-CD3 stimulated A decrease followed T-cell blastogenesis in intracellular calcium ion concentration and blood pressure in spontaneously hypertensive rats, but not in normotensive rats treated with MO (Attakpa et al., 2017).

Conversely, a study by Randraiamboavonji et al. showed that MO seed powder given for 8 weeks does not affect blood pressure in a spontaneously hypertensive rat model. However, it provides a cardioprotective effect by reducing nocturnal heart rate increasing ejection volume and cardiac output (Randriamboavonjy et al., 2016). Another study by Ofem et al. (2015) reported that aqueous MO leaf extracts might resolve the abnormalities in hematology parameters in rats treated with a high-salt diet such as white blood cells, red blood cells, platelet counts, packed cell volume, lymphocytes, platelet large cell ratio, mean platelet volume, and platelet distribution width (PDW).

#### 3.2.5 Protective Effects on Target Organ Damage due to CMD

Studies proved that diabetes mellitus, hypertension, and metabolic disorders raised cardiovascular problems by inducing injury to several organs. The most common damage caused by CMD are cardiac, renal dan vascular disorders (Cea-Calvo et al., 2006; Oladapo et al., 2012; Abegaz et al., 2017).

Variety of stimuli in cardiometabolic diseases, such as pro-inflammatory cytokines, oxidative stress, or DNA damage in cardiometabolic conditions, trigger apoptosis which eventually causes several organ damages (Lee and Gustafsson, 2009). The antiapoptotic effect of MO is one of the essential features of MO that provides protective effects to organ damage due to CMD.

MO seed provided cardioprotective effects in myocardial infarction in mice model. Next, MO was demonstrated to increase the survival rate of left ventricular ejection fractions and prevent cardiac remodeling by reducing myocardial apoptosis. Alleviation of myocardial apoptosis was marked with reduction of TUNEL positive cells and Bax gene expressions (pro-apoptotic) and increased BCl2 (antiapoptotic) expressions (Li Y. J. et al., 2020). Similarly, Khalil et al. (2020) reported cardioprotective effects of MO leaves by downregulating Bax, caspase-3, Apaf1, p53, upregulation of BCl-2, and increased activity in antioxidant enzymes. Several studies using extracts from leaves, seeds, and isolated compounds reported similar cardioprotective activity, which was mediated by antioxidant effects of MO (Nandave et al., 2009; Cheraghi et al., 2017; Bitrus et al., 2018; Gouda et al., 2018; Vera-Nunez et al., 2021).

Similarly, protective effects of MO in other target organs, including liver and kidney, were mainly facilitated by antioxidant, anti-inflammatory, or antiapoptotic mechanisms. Soliman et al. (2020) reported that MO leaf extract restored liver and renal histopathology architecture by reducing caspase-9 and BCl2 expression in the liver and renal. Adel-Daim showed that MO methanol extract attenuated histopathological features of the liver by increasing antioxidant enzymes, reducing TNF-α, IL-1β, NF-κB, downregulating iNOS, and inhibiting pro-apoptotic proteins (Abdel-Daim et al., 2020). In a study by Das et al. (2012) in high-fat diet-induced hyperlipidemia, MO leaves ethanol extracts provided prevention of liver damage and inhibited fatty liver progression, mainly by increasing GSH.

MP seed powder given for 8 weeks was shown to provide cardio- and vasculoprotection in spontaneously hypertensive rats via upregulation of PPAR-α and increasing plasmatic prostacyclins (Randriamboavonjy et al., 2016). When given for 20 weeks, the same research group showed that MO seed powder was found to reduce free 8-isoprostane and SOD2, vascular p22phox and p47phox, iNOS, and NF-KB and enhance endothelium-dependent carbachol-induced relaxation (Randriamboavonjy et al., 2017). Chen et al. (2012) demonstrate that MO leaves hexane extracts lower pulmonary arterial pressure and the thickening of vessel walls in monocrotaline-induced pulmonary hypertension in rats.

Several studies used MO topically as wound healing in a diabetic rat model. The studies reported that wound area was reduced, followed by enhancement of tissue regeneration and downregulation of inflammatory mediators (TNF-α, IL-1β, IL-6, iNOS, MCP-1, and COX-2) (Muhammad et al., 2016; Azevedo Í et al., 2018; Chin et al., 2019). Interestingly, Muhammad provided additionally *in vitro* data that support the antibacterial activity of MO in *Staphylococcus aureus*, *Pseudomonas aeruginosa*, and *E. coli*. Their findings support the use of MO in diabetic ulcers that are commonly infected by bacteria.

#### 3.2.6 Gut Microbiome Modulatory Effects

Over the past years, gut microbiomes have been studied concerning various aspects of human health, including CMD. Changes in the function and composition of gut microbiota have been linked to the theory of aging, dysregulation of immune system age-related diseases such as coronary artery disease, diabetes, and cancer (Kazemian et al., 2020). Several studies proposed dysbiosis and the leaky gut concept to contribute to CMD development (Witkowski et al., 2020; Jansen et al., 2021). Research provided data that MO extracts promote gut and intestinal tissues integrity (Wang et al., 2019b; Tian et al., 2021).

In addition to its active compounds with antimicrobial activities such as kaempferol and isoquercitrin, amino acids and peptides, and lectins in MO were thought to be the primary modulators of gut microbiomes composition (Xiao et al., 2020). Crosstalk between protein/amino acid from diets may alter gut microbiomes’ profiles and functions, further impacting the host’s metabolism (Zhao et al., 2019; Wu et al., 2021). Nutritional analysis of MO indicates that MO leaf extracts were rich in protein content, ranging from 23–30% and low fat (Sultana, 2020). Further analysis of MO protein isolates demonstrated about 42–25% of the total proteins contain essential amino acids (threonine, valine, methionine, isoleucine, leucine, phenylalanine, histidine, lysine, arginine, and tryptophan (Aderinola et al., 2018).

Next, Tian et al. investigated the effects of MO polysaccharides (MOP) in C57BL mice. They found that MOP reduced plasma glucose and total cholesterol and improved oxidative markers (MDA, SOD, and catalase). All the metabolic improvements were due to the action of MOP in changing villi length and crypt depth in ileum and jejunum, thereby increasing beneficial and reducing harmful bacteria in the caecum. Tian et al. (2021) reported that MOP regulates metabolites in various micro-and macromolecules. Caecal enzymes, namely 1-phosphofructokinase and beta-fructofuranosidase, were similarly impacted by MOP. Both enzymes exhibit an essential role in carbohydrate homeostasis (Tian et al., 2021).

The effect of MO polysaccharides (MOP) was studied by Wang et al. and proved that MOP lowered serum TNF-α and diamine oxidase. MOP improves gut microbiota composition by ameliorating intestinal tissue integrity and increasing the production of SCFA and lactic acid. The activity of gut amylase, lipase, and alkaline phosphatase was also found to be raised by MOP (Wang et al., 2019b). Yet, in a study done by Villarruel-Lopez et al. (2018), powder extract of MO leaves provides a hypoglycemic effect but did not change the number of lactic acid bacteria. Reduction of colon pH and increased SCFA and gut microbiota by MO leaves extracts were also demonstrated in an *in vitro* model of the gastrointestinal system (Dou et al., 2019). The major bioactive compounds of MO released during the digestion process were 6,8-di-*C*-glucosylapigenin, catechin, quercetin-3-O-β-D-glucoside, and ferulic acid (Dou et al., 2019).

Another study by Gao et al. studied the effect of MO leaves aqueous extracts and found that 4-week supplementation of MO in healthy mice caused a slight reduction in plasma glucose and lipid profiles. However, these changes were followed by mild activation of inflammatory response in the gut and liver changes in gut barrier function. Alteration in the gut inflammatory responses was correlated with the changes in gut bacteria composition such as *Firmicutes*, *Eubacterium rectale*/*Clostridium coccoides* group, segmented filamentous bacteria, and *Enterococcus* spp. In their study, Gao et al. did not specify the ratio or the number of gastrointestinal microbes. Researchers showed that altered number and functions of bacteria were found in the digestive tracts of mice fed high-salt or low-salt food. This was done by analyzing the bacteria’s mRNA in the mice (Gao et al., 2017).

#### 3.2.7 Toxicity Studies

*In vitro* studies in human pulmonary artery endothelial cells (HPAEC) (Aekthammarat et al., 2020a), RAW364.7 cells (Tan et al., 2015; Cui et al., 2019), HaCaT and J774A.1 cells (Liao et al., 2018), human MDM (Luetragoon et al., 2020), human PBMC (Oldoni et al., 2021), HepG2 cells (Sangkitikomol et al., 2014), LO2 cells (Sun et al., 2019) showed that MO was not cytotoxic at the pharmacological dose used. However, one study by Araújo et al. (2013) showed that coagulant MO lectin (cMol) from seed aqueous extracts were potentially cytotoxic in PMBC, while the diluted extracts were not.

Most animal studies did not provide toxicity results. However, in several studies that reported acute and chronic toxicity studies, lethal doses were found at a relatively high dose (up to 10–20 times of the adequate amount used in animals) (Jaiswal et al., 2009; Araújo et al., 2013; Adedapo et al., 2015; Waterman et al., 2015; Atta et al., 2017; Sun et al., 2019). Acute dermal toxicity study of MO as a wound dressing in diabetic ulcer model did not cause any toxicity on the skin (Chin et al., 2019). A study performed by Gao et al. (2017) found that aqueous extract of MO causes mild abnormal activities in the gut and liver after 4-week supplementation, which caused increased inflammatory responses in the gut and liver and compromised gut barrier function.

### 3.3 Effects of MO From Human Studies

Out of the seven manuscripts reporting the effects of MO in humans, four studies were done on type 2 diabetes mellitus subjects, one study in overweight or obese subjects, one study on hypercholesterolemia, and one study in healthy volunteers. All the studies were done on a limited number of patients. Trials done by Sandoval and Jimeno (2013) included the highest number of patients (total 68 patients, 33 in MO groups and 35 in the placebo group, given for 30 days in high LDL-c adult subjects. However, all seven clinical studies used different doses, making further evaluation more complicated. Only five out of the seven studies included the method of drug preparation in the manuscripts. Four of the studies used dried powdered leaves filled into capsules with no further extraction method. Yet, the study by Ezzat used powdered MO leaves macerated with 70% ethanol, with a 10% yield.

Anthanont et al. provided preliminary safety studies in healthy volunteers up to 4 g single dose of MO leaf powder. They demonstrated that the highest amount (4 g) significantly increased insulin secretion (Anthanont et al., 2016). In addition, a single dose study in non-diabetic vs. diabetic subjects administered with 20 g MO leaf powder added to a meal resulted in the lower increment of postprandial blood glucose in T2DM (Leone et al., 2018).

Moreover, Ifeoma demonstrated the effect of steamed MO leaves, 0–60 g/day for 14-days in a clinical trial. The study was done in 32 subjects with six subjects per group. Steamed MO leaves consumed together with meal gave no difference in waist circumference, waist-hip ratio, and FPG in all groups. However, the groups that received 40–60 g per day resulted in the reduction of SBP (but not DBP), followed by an increase in TG, LDL, and HDL (Ifeoma, 2020). Another study in T2DM by Dominguez-Rodriguez et al. reported the beneficial effect of MO leaves extract after 10 weeks of treatment. The treatment resulted in a reduction in BMI, insulin, blood pressure, and an increase in HDL (Dominguez-Rodriguez et al., 2016). However, a study in therapy naïve T2DM patients for 4 g/day for 4 weeks does not affect glycemic control (Taweerutchana et al., 2017).

A study by Ezzat et al. in overweight/obese subjects reported that 400 mg leaves ethanol extract in a capsule given for 8 weeks resulted in a significant reduction in BMI total cholesterol and LDL vs baseline. In their report, Ezzat TC explained the mechanism of action by their nonclinical study in high-fat diet-induced obesity in rats. Their study mentioned MO reduced leptin, increased adiponectin, and suppressed HMG-CoA reductase activity (Ezzat et al., 2020). Yet, Sandoval and Jimeno (2013) reported that 2,100 mg/day MO powdered leaf capsules per day administered for 30 days resulted in a similar LDL-c reduction vs. placebo.

None of the seven studies on human subjects reported any significant adverse events. Anthonont et al. reported no adverse events when MO leaf powder capsules were given to healthy subjects up to 4 g for 2 weeks. In the dose-escalation study, where the subjects received an oral dose of MO once daily in increments of 0 (baseline), 1, 2, and 4 g for 2 weeks, Anthonont et al. (2016) recorded no change in the volunteers’ BUN, Cr, AST, ALT, and plasma glucose levels. Out of the seven trials, the most prolonged study period was done on obese type 2 diabetics subjects treated with metformin. Ten weeks of treatment using MO leaves extracts does not result in increased adverse events compared to the control group.

## 4 Discussion

**FIGURE 2:**
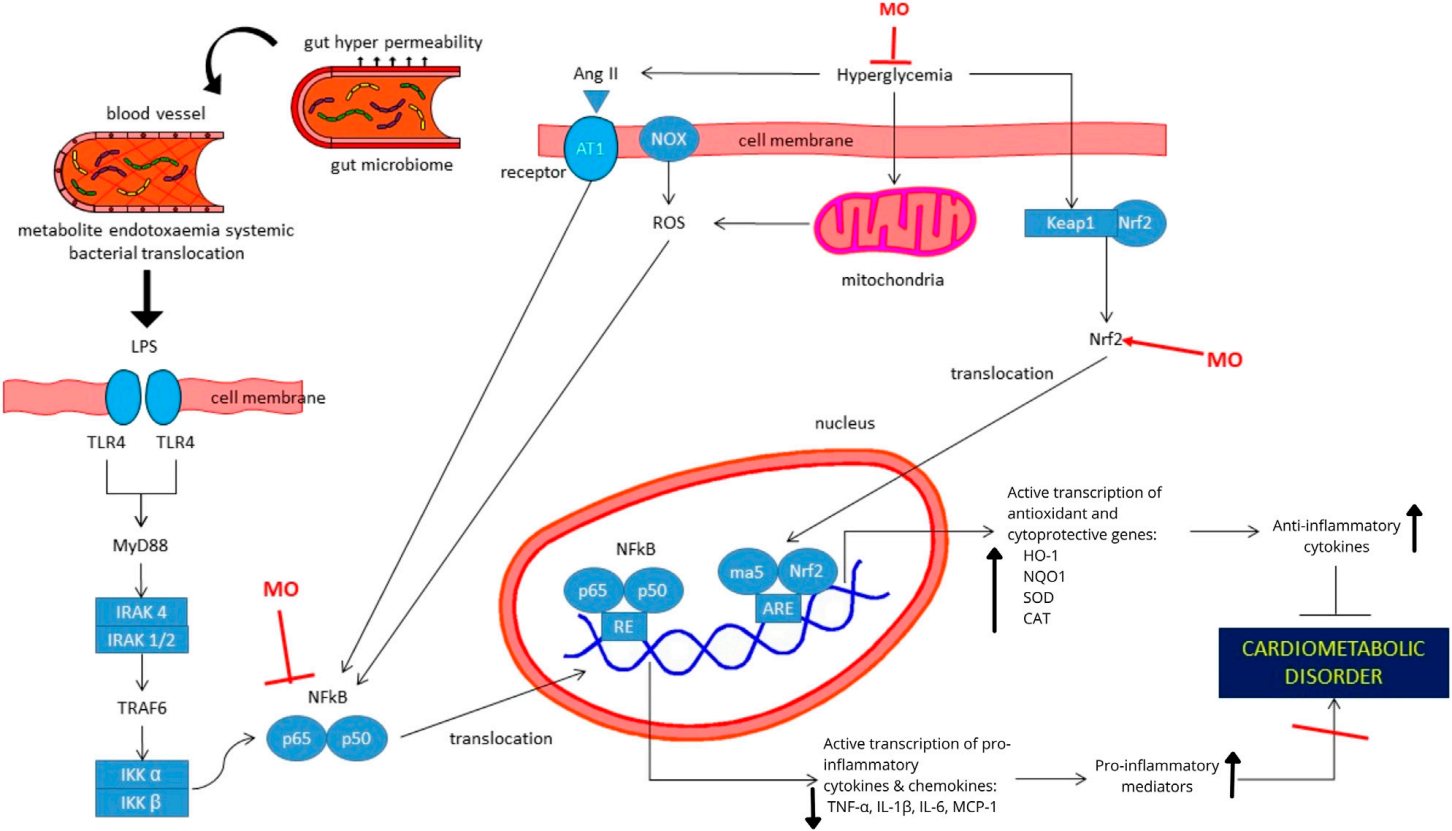
Proposed mechanism of actions of Moringa oleifera extracts or its isolated compounds for the treatment of cardiometabolic-related disorders. Antioxidative and anti-inflammatory activities of MO are mainly responsible for its action in hyperglycemia, hypertension, dyslipidemia and obesity. MO upregulates Nrf2, which resulted in the increased transcription of antioxidative and cytoprotective genes. Further, the process increases anti-inflammatory cytokines. In addition, MO provides its anti-inflammatory effects by suppressing NF-kB protein and its translocation to the nucleus, which resulted in downregulation of pro-inflammatory genes. Suppressive effects of MO to NF-kB can be resulted from direct downregulation in NF-kB or indirect effect due to the change in intestinal tissue integrity and gut microbiome composition and functions. Abbreviation: MO, Moringa oleifera; Ang-II, Angiotensin II; AT1, Angiotensin 1; CAT, catalase; HO-1, Heme oxygenase-1; IKKα, Inhibitory kB kinase alpha; IKKβ, Inhibitory kB kinase beta; IRAK, Interleukin-1 receptor associated kinase; Keap1, Kelch-like ECH associated protein 1; LPS, lipopolysaccharides; MCP-1, Monocyte chemoattractant protein; MyD88, Myeloid differentiation primary response protein; NOX, NADPH-oxidase; NQO1, NAD(P)H quinone dehydrogenase; Nrf2, Nuclear factor erythroid 2-related factor 2; ROS, Reactive oxygen species; SOD, sodium dismutase; TLR4, Toll-like receptor 4; TNFα, Tumor Necrosis Factor-Alpha.

Numerous nonclinical studies provided the beneficial effects of MO for the treatment of CMD-related diseases, including antihyperglycemic, antihyperlipidemic, antihypertensive, anti-inflammatory, and the modulation of gut microbiota. We summarized the possible mechanism of MO actions in Figure 2.

The actions of MO in CMD-related disorders are mainly due to its anti-inflammatory and antioxidative effects. Anti-inflammatory effects of MO are provided by suppressing NF-kB protein, which is activated by increased blood pressure, blood sugar, oxidative stress, and translocation of bacteria from the gastrointestinal tract to the blood vessels. Next, the above activity prevents the translocation of transcription factors to the nucleus so that MO can inhibit the transcription of inflammatory mediators. In addition, MO upregulates Nrf2, which resulted in the increased transcription of antioxidative and cytoprotective genes. Further, this increases anti-inflammatory cytokines. Antioxidative and anti-inflammatory activities of MO are mainly responsible for its action in hyperglycemia, hypertension, dyslipidemia, and obesity. Thus, MO can prevent the development of cardiometabolic disorder.

Despite the fact that many studies have been conducted to investigate the effect of MO on CMD, the method of extract preparation and dose used varied. The majority of studies utilized the MO dose in a CMD rodent model within the range of 200–800 mg/kg BW, or approximately 32–128 mg in a 60-kg human. However, whether the right dose can be extrapolated to humans, the use of standardized preparations with a dose-finding study is still needed (Reagan-Shaw et al., 2008; Nair et al., 2018).

The amount of MO used in clinical trials was similarly varied. Some of the studies used a relatively high dose. The high dosage used might be explained by the direct use of dried MO leaves filled into capsules with no further extraction method. With the difficulties in providing the proper mode of extractions and its dose translation for application in the clinical trial, we propose that several bioactive compounds studied, with potent pharmacological activity such as isothiocyanates (Waterman et al., 2015; Huang et al., 2020) niazirin (Wang et al., 2019a; Bao et al., 2020), β-sitosterol (Liao et al., 2018), N,α,L-rhamnopyranosyl vincosamide (Panda et al., 2013; Cheraghi et al., 2017), 1-O-(4-hydroxymethylphenyl)-α-L-rhamnopyranoside (MPG) (Sun et al., 2019), may be used for further study in humans rather than in extract form.

Though numerous studies have been studied on the benefit of MO in various animal models of cardiometabolic-related disorders, clinical trials recorded up to date were scarce. The present review presented seven studies for the use in cardiometabolic-related diseases and found that preparations of MO, the dose used, study design, duration, and outcomes are varied. All the clinical trials were done in a limited number of subjects (ranged from 10 to 68 subjects in seven studies). Despite the limited number of subjects in clinical trials, studies suggested that MO might demonstrate dose-effect relationships in providing glycemic control, lowering blood pressure, and reducing BMI (Anthanont et al., 2016; Ifeoma, 2020).

Yet, researchers must carefully judge the precision, accuracy, and robustness of clinical trial methods used. Some studies with positive results used a high dose of MO (up to 4 g of MO leaf power), making the mode of administration in capsules somewhat problematic. In their study, Leone et al. added MO powder to a meal. However, the poor taste of MO powder resulted in poor acceptability (Leone et al., 2018).

Regarding the safety profile, toxicity studies in the animals provided a convincing result, with a relatively high dose of LD50, ranging from 2,000 to 5,000 mg/kg BW (Jaiswal et al., 2009; Araújo et al., 2013; Adedapo et al., 2015; Waterman et al., 2015; Atta et al., 2017; Sun et al., 2019). Up to date, clinical studies provided preliminary data on the potential adverse events—no notable increase in adverse events when given up to 4 g. However, a study using a higher dose (40–60 g) potentially caused changes in routine hematology parameters and increased cholesterol levels.

The present review had several limitations that can be highlighted. Firstly, the variability of the reporting method of the manuscripts in terms of the steps in extraction and purification. Detailed methods provided are very important to provide reproducibility of the results. To date, there has not been a single consensus agreed upon as guidance to report phytopharmacological or ethnopharmacological research. Heinrich et al. (2020) recently published a guidance for researchers to report pharmacological studies of bioactive preparations. However, our present systematic review did set a time limit for the studies included, which might be the reason that only 40 out of 108 of the included studies reported the yield and contents of their extracts. The other limitation is the lack of long-term studies in animals or in clinical trials. Most pharmacological treatments for CMD require long-term treatments. Thus, research with an appropriate study duration is still warranted to confirm the benefit of MO in CMD.

In conclusion, various animal models provided robust beneficial effects for the use of MO for the treatment of CMD. Available toxicity studies *in vitro* and in animals demonstrated high safety. Yet, up to date, well-conducted clinical trial data that applies standardized preparations and doses of MO extracts, particularly in T2DM and obese subjects, are still needed to confirm the efficacy and safety in patients.

## Data Availability

The original contributions presented in the study are included in the article/Supplementary Material, further inquiries can be directed to the corresponding author.
