# Supplementary material for: Moringa Oleifera Lam. in Cardiometabolic Disorders: A Systematic Review of Recent Studies and Possible Mechanism of Actions
Source: Front Pharmacol. 2022 Mar 30;13:792794. doi: 10.3389/fphar.2022.792794 (PMC9006177; doi:10.3389/fphar.2022.792794)
Supplement: Supplementary file 1 [file DataSheet1.pdf]

Supplementary Material

Moringa oleifera in cardiometabolic disorders: A systematic review of recent studies and possible mechanism of actions

Melva Louisa[1*], Cyntia Gracesella Hutami Patintingan[2], Bantari WK Wardhani[3]

[1] Department of Pharmacology and Therapeutics. Faculty of Medicine, Universitas Indonesia, Jakarta, Indonesia

[2] Master Program in Biomedical Sciences, Faculty of Medicine, Universitas Indonesia, Jakarta, Indonesia

3 Department of Pharmacology and Therapeutics, Faculty of Military Pharmacy, Indonesia Defense University, West Java, Indonesia

***Correspondence:**

Corresponding Author

melva.louisa@gmail.com

Table of Contents

**Supplementary Material**	**Page**
Table S1. Search terms used in the study	3
Table S2. Nonclinical studies of *Moringa oleifera*	4
Table S3. Clinical Studies of *Moringa oleifera*	27
References	29

Table S1. Search terms used in the study

Database	Search Term
PubMed	TITLE/ABS Moringa oleifera AND ((cardiovascular diseases [MeSH]) OR (Metabolic Diseases [MeSH]) OR (Blood Glucose [MeSH]) OR (Obesity [MeSH]) OR (hypertension [MeSH]) OR (Kidney Failure [MeSH]) OR (Molecular Mechanisms of Pharmacological Actions [MeSH]) OR (Pharmacological and Toxicological Phenomena [MeSH]) OR Oxidative Stress [MeSH]) OR (Inflammation [MeSH]) OR (Gastrointestinal Microbiome [MeSH]) OR (Immune System [MeSH])
Scopus	(TITLE-ABS-KEY (moringa AND oleifera) AND TITLE-ABS-KEY (((cardiovasc*) OR (cardiac) OR (cvd) OR (ischemi*) (metaboli*) OR (lipid) OR (cholesterol) OR (ldl) OR (hdl) OR (tg) OR (tryglyceride*) OR (diabetes) OR (dm) OR (dmt2) OR (insulin) OR (hyperglyc*) OR (hypoglyc*) OR (glucose) OR (kidney) OR (hypertens*) OR (blood AND pressure) OR (diuretic) OR (pharmacolog*) OR (toxic*) OR (oxidative AND stress) OR (antioxidant) OR (inflam*) OR (microbiome) OR (microbiota) OR (immune))))
CENTRAL	#1 Moringa oleifera<br>#2 MeSH descriptor [cardiovascular diseases] explode all trees<br>#4 MeSH descriptor [Metabolic Diseases] explode all trees<br>#5 MeSH descriptor [Blood Glucose] explode all trees<br>#6 MeSH descriptor [Obesity] explode all trees<br>#7 MeSH descriptor [Hypertension] explode all trees<br>#8 MeSH descriptor [Kidney Failure] explode all trees<br>#9 MeSH descriptor [Oxidative stress] explode all trees<br>#10 MeSH descriptor [Inflammation] explode all trees<br>#11 MeSH descriptor [Gut microbiome] explode all trees<br>#12 MeSH descriptor [Immune system] explode all tree<br>#13 insulin<br>#12 glucose<br>#13 lipid<br>#14 cholesterol<br>#15 diuretic<br>#1 AND {#2 - #15}

Table S2. Nonclinical studies of *Moringa oleifera*

Author, year	Type of extracts/ main bioactive compound used	Methods of extracts/ bioactive preparation	Yield of preparation from raw material (wt/wt)	*In vitro*/ *in vivo* models used	Treatment/ duration	Effects	Mechanism	Toxicity
Abarikwu, 2017 [1]	MO seed oil	Cold pressed from freshly shelled moringa seeds	Not reported	HgCl2-induced hepato- and nephrotoxicity in Male Wistar albino rats	1.798 mg/kg BW p.o. three times per week for 21 days.	- Amelioration of kidney and liver injury<br>- ↓ MDA, SOD, CAT, GST, GSH.<br>- normalization of creatinine, bilirubin GGT, LDH, ALP	Antioxidant	No toxicity was observed
Abd El Latif, 2014 [2]	MO leaves aqueous extracts	Dried water extract from fresh green leaves MO. Reconstitution were done in distilled water prior to administration	22%	Alloxan-induced diabetes in rats	250 mg/kg BW p.o for 18 days	- Improvement of blood glucose, TG, cholesterol, and MDA levels.<br>- Normalization of hepatic pyruvate carboxylase mRNA expressions.<br>- ↑ PC in hepatic tissues.<br>- ↑ body weight<br>- restoration of liver FAS mRNA expression<br>- Improved histological structure of the liver and pancreas.	Glucose metabolism, lipid metabolism, hepatoprotective	Not reported
Abd Eldaim, 2017 [3]	MO leaves aqueous extract	Dried water extract from fresh green leaves MO	Not reported	Alloxan-induced diabetes in Wistar albino rats	250 mg/kg BW p.o for 18 days	- Protective effect on hepatic and pancreatic tissues<br>- Normalized hepatic GSH, SOD, CAT, and glycogen synthase gene expression of glycogen synthase.<br>- ↓ blood glucose<br>- ↓ hepatic lipid peroxidation,	Antioxidant, glucose metabolism, antiapoptotic, hepatoprotective	Not reported

						- ↓ PC and caspase 3 gene expression.		
Abd Rani, 2019 [4]	MO ethanol extract and its isolates	Leaves, seeds, and pods were macerated separately. Fractionation were done sequentially using hexane, ethyl-acetate, and acetone.	Leaves: 5% Seed: 3.3% Pod: 3%	RBL-2H3 cells	7.81, 15.62 and 31.25 µg/mL	All extracts and isolated compounds significantly inhibited beta-hexosaminidase, histamine, and cytokine (IL-4 and TNF-α) release.	Anti-inflammation	Cytotoxicity test: at 7.81 - 31.25 µg/mL, extracts and isolates were found not cytotoxic.
Abd-Elhakim, 2021[5]	MO ethanol extract	Hydro distillation techniques with ethanol	Not reported	Melamine-induced hepatorenal impairment in male S.D. rats	- 800 mg/kg BW/day p.o for 2 weeks as prophylaxis.<br>- 800 mg/kg BW/day p.o. for 2 weeks, co-treated with melamine	- ↑ CAT, GPx, and BCl-2 expression<br>- ↓ p53 expression.<br>- Suppressed expression of the proinflammatory cytokine, KIM-1 and TIMP-1.<br>- Improved hepatorenal efficacy when given simultaneously with melamine.	Antioxidant, anti-inflammation.	Not reported
Abdel Fattah, 2020 [6]	MO leaves aquatic extract	Dried MO from fresh leaves macerated in cold water, boiled for 15 min, and then filtered.	Not reported	Lead acetate–induced liver injury in male Wistar rats	200 mg/kg BW p.o for 4 weeks	- Amelioration of weight gain reduction<br>- ↓ TC, TGs, LDL-C AST, ALT, ALP, TNF-α.<br>- ↑ in HDL-c, TP, albumin, GSH, and SOD.<br>- ↓ DNA fragmentation<br>- improved hepatic lesions and tissue score damage.	Antioxidant, lipid metabolism, hepatoprotective.	Not reported

Abdel-Daim, 2020 [7]	MO leaves methanol extract	Powdered leaves MO were extracted using methanol 70%.	Not reported	Lead acetate–induced liver injury in male Wistar rats	250 mg/kg BW i.p for 14 days	- ↓ in relative kidney weight, urea, and creatinine.<br>- Attenuation of abnormal histopathological features<br>- ↑ CAT, GPx1.<br>- ↓ TNF-α, IL-1β, and NF-κB<br>- Downregulation of iNOS.<br>- Inhibiting the pro-apoptotic proteins and enhancing the antiapoptotic protein	Antioxidant, anti-inflammation, anti-apoptotic	Not reported
Abdel-Daim, 2020 [8]	MO leaves ethanol extract	Fine powdered leaves MO was extracted using ethanol absolute.	7.8%	Cobalt-induced renal oxidative damage and inflammatory injury in male S.D rats.	Prophylaxis: 400 mg/kgBW/ day for 2 weeks alone, 4 weeks with cobalt<br><br>Treatment: 400 mg/kgBW/day for 4 weeks with cobalt, then for 2 weeks alone.	- Improved body weight gain.<br>- ↓ urea, creatinine, MDA, and 8-OHdG.<br>- ↑ globulin, SOD, CAT, GSH.<br>- ↓ CRP, MPO, TNF-α, and NO.<br>- Down-regulation of NF-kB and IL-6.<br>- More effective when + CoCl2 as a prophylactic regimen.	Antioxidant, anti-inflammation	Not reported
Abdou, 2018 [9]	MO leaves ethanol extract	MO was macerated in ether for 72 h, and then evaporated. The residue was further macerated in ethanol and then freeze dried	Not reported	TiO_2 nanoparticles-induced nephrotoxicity in male albino rats.	400 mg/kgBW for 60 days	- ↓ creatinine, urea, uric acid, renin, MDA.<br>- ↑ SOD, GST, GSH, GPx, total thiols.<br>- ↓ TNF-α, NF-κB, HSP70.<br>- Upregulation of Nrf2 and HO-1 mRNA.<br>- Downregulation of KIM1 mRNA.	Antioxidant, anti-inflammation	Not reported

Abou-Zeid, 2021 [10]	MO leaves ethanol extract	Powdered leaves MO was extracted using ethanol absolute.	7.8%	Tilmicosin-induced renal damage in male S.D. rats.	400 and 800 mg/kg BW for 7 days	- ↓ creatinine, urea, sodium, potassium, and GGT activity<br>- ↑ TP and albumin<br>- ↓ renal tissue H2O2 and MDA<br>- ↑ SOD, GPx<br>- ↓ TNF-α, IL-1β<br>- ↓ Desmin, nestin and vimentin mRNA expression<br>- Histopathological alterations in renal glomeruli, tubules, and interstitial tissue.<br>- ↓ in frequencies and severities of nephropathy.	Antioxidant, anti-inflammation, nephroprotective	Not reported
Abu-Zeid, 2021[11]	MO leaves ethanol extract, and MO leaves extract-Selenium nanoparticles	● Powdered leaves MO was extracted using ethanol absolute.<br>● Filtered suspension of dried leaves MO was mixed with deionized water, selenious acid, ascorbic acid subsequently.<br>● It forms a SeNPs nano-solution of MO leaves.	7.8%	Melamine induced nephrotoxicity in male S.D. rats	800 mg/kg BW extract and 200 µg mg/kg BW nanoparticle extract daily for 28 days.	- ↓ creatinine, urea, BUN.<br>- ↑ TAC, GSH, SOD, CAT, GPx, MDA, PCO, TNF-α.<br>- Down-regulation of Bax, Caspase-3, FasL mRNA expression.<br>- Up-regulation of Bcl2, PCNA, and Ki-67.<br>- ↓ in severity and frequency of nephrotoxic lesions but did not show the reestablishment of normal histology.	Anti-inflammation, antioxidant, Antiapoptotic, nephroprotective	No signs of toxicity.
Adedapo, 2015 [12]	MO food supplement extracted	Powdered leaves of MO was extracted using methanol.	9.6%	Carrageenan-induced paw edema, histamine-	50, 100 and 200 mg/kg BW	- ↓ size of edema, the number of writhes, licking time, and frequency.	Analgesic, anti-inflammation, antioxidant.	Acute toxicity test:<br>- 200, 400, 800 mg/kg BW:

	with methanol			induced pedal edema, acetic acid-induced pain, formalin-induced pain, in female white Wistar strain albino rats		- Dose-related free radical-scavenging property		slightly dull and were fully recovered after 48h, no death<br>- 1600 mg/kgBW: dull, severe lethargy. 2 out of 5 died.<br>- 3200 mg/kg BW: Severe lethargy, rough hair coat. 4 out of 5 died.
Adepoju-Bello, 2017 [13]	MO leaves water or ethanol, or methanol extract	Cold-maceration using five different solvents: (1) 100% Methanol, (2) 100% Ethanol, (3) Water-Methanol (50/50), (40 Water- Ethanol (50/50) and (5) 100% Water	(1) 2.49 %<br>(2) 3.17 %,<br>(3) 9.42 %,<br>(4) 8.52 %<br>(5) 6.47 %.	Alloxan-induced diabetes in male and female Wistar rats	200 - 400 mg/kg BW once daily for 24 days.	- ↓ blood glucose levels.<br>- 50% ethanolic extract at 300 mg/kg showed superior antioxidant properties, weight restorative, and pronounced hypoglycemic effects.	Glucose homeostasis, antioxidant	Not reported.
Aektham marat, 2019 [14]	MO leaves aqueous extract	Air-dried powdered leaves MO were extracted in distilled water.	4.975%	L-NAME-induced hypertension in Male Wistar rats	30 and 60 mg/kg BW once daily for 3 weeks.	- ↓ BP and tachycardia.<br>- ↓ impairment of acetylcholine-induced relaxation<br>- ↓ hyperreactivity of adrenergic-mediated contraction.<br>- ↓ vascular O_2 production<br>- ↓ MDA, SOD, CAT	Antihypertensive, vasculoprotective, antioxidant	Not reported.

Aektham marat, 2020 [15]	MO leaves aqueous extract.	Air-dried powdered leaves MO were extracted in distilled water.	4.975%	Mesenteric arterial beds isolated from L-NAME-induced hypertension in male Wistar rats	0.001–3 mg in 0.1 mL injection volume	- Relaxation in methoxamine pre-contracted arterial beds.<br>- Inhibitory effect on $CaCl_2$ and phenylephrine-induced contractions.	Vasculoprotective	Not reported.
Aektham marat, 2020 [16]	MO leaves aqueous extract.	Air-dried powdered leaves MO were extracted in distilled water.	4.975%	L-NAME-induced hypertension in male Wistar rats<br><br>Human pulmonary artery endothelial cells (HPAEC).	Rats: 1–30 mg/ kg BW<br><br>HPAEC: 3–30 µg/mL.	- ↓ in MAP when administered alone.<br>- Induced relaxation in methoxamine pre-contracted mesenteric arterial beds.<br>- Induced NO production in HPAEC.	Vasculoprotective	Cytotoxicity test: At 0.1–300 µg/ mL did not significantly influence the cell viability. The viability was decreased at the concentrations of 1000 and 3000 µg/mL.
Aju, 2019 [17]	MO leaves methanol extract.	Dried leaf powder was serially extracted with petroleum ether, chloroform, and methanol.	Not reported	Streptozotoc in- induced diabetes in male albino S.D. rats	300 mg/kg BW once daily for 60 days	- ↓ blood glucose and HbA1c, TBARS, HP, and conjugated dienes (CD).<br>- ↑ plasma insulin, SOD, CAT, GPx, GR, GSH.<br>- Improve the histopathology of the diabetic heart.	Glucose homeostasis, antioxidant	Not reported
Akinrinde, 2020 [18]	MO leaves methanol extract.	Air-dried powdered leaves MO were extracted sequentially in n-hexane and two	Not reported	Ischemia reperfusion-induced acute kidney injury in Wistar rats.	200 and 400 mg/kg BW for 7 days before IR induction	- ↓ BUN, creatinine, NO, MDA, AOPP, carbonyls.<br>- Enhancement of thiols and GSH levels, GPx, and GST activities.	Antioxidant, Nephroprotective	Not reported

		times of methanol-water (80:20).				- Improvement in renal histology.		
Al-Malki, 2015 [19]	MO seed powder.	Not reported	Not reported	Streptozotocin-induced diabetes in male albino rats.	50 and 100 mg/kg BW in food for 4 weeks.	- ↓ water consumption, fasting blood sugar, MDA, IgG, IgA, IL-6, HbA1c, α-amylase, BUN, uric acid, creatinine, sodium, and potassium.<br>- ↓ food intake, body weight gain, food efficiency ratio (FER).<br>- ↑ CAT, SOD, GSH<br>- Restored the kidney and pancreatic tissue of diabetic rats to normal.	Glucose metabolism, antioxidant, Nephroprotective	Not reported
Albasher, 2020 [20]	MO leaves methanol extract.	Fine powdered leaves MO was extracted using 70% of methanol.	14.3%	Lead-induced hepatotoxicity in male Wistar rats.	250 mg/kg BW p.o for 14 days	- ↓ Pb(II) concentration<br>- ↓ ALT, AST, MDA, NO, TNFα, IL-1β levels, NF-κB p65 level, and iNOS mRNA expression.<br>- ↓ Bax and caspase-3, and ↑ in Bcl-2 levels.<br>- ↑ GSH, SOD, CAT, GPx, and GR.<br>- Prevention of tissue injury.	Antioxidant, anti-inflammation, antiapoptotic, hepatoprotective	Not reported
Alhakmani, 2013 [21]	MO flowers ethanol extract.	Powder of MO flowered were macerated in 70% ethanol.	8.69%	Egg albumin	100-500 µg/mL	- Dose-dependent scavenging activity<br>- inhibition of denaturation of egg albumin.	Anti-inflammation, antioxidant	Not reported

Anudeep, 2016 [22]	MO seeds	Kernels were obtained from matured and dried MO seeds.	6.5%	Splenocytes from male Swiss albino	0.01 to 10 µg	- ↑ proliferation of splenocytes at low concentration<br>- ↑ NO production up to 6-fold.	Anti-inflammation	Not reported
Araújo, 2013 [23]	MO seeds aqueous extract, cMoL (coagulant MO lectin); WSMoL (water-soluble MO lectin)	Crushed MO seeds were extracted in distilled water.	Not reported	Carrageenan-induced pleurisy in male Balb/c mice<br><br>Peritoneal macrophages isolated from LPS-stimulated male Balb/c mice.	Seed extract: 125, 250 or 500 mg/kg<br><br>Seed extract (6.25–50 µg/mL), diluted seed extract (50–400 µg/mL) or cMoL or WSMoL (6.25–100 µg/mL)	- ↓ Leukocyte migration, myeloperoxidase activity, NO, TNF-α and IL-1β.<br>- ↓ number of leukocytes in the lungs.	Anti-inflammation	Seed extract and cMoL are potentially cytotoxic for PBMCs, while the diluted seed extract and WSMoL are not cytotoxic to these cells. Acute toxicity test (2000 mg/kg) showed no signs of systemic toxicity
Arise, 2016 [24]	MO flowers ethanol extract	Air-dried MO from fresh flowers were pulverized and extracted in 95% ethanol.	Not reported	Streptozotocin-induced diabetes in male albino rats.	100, 200, 300 mg/kg BW once daily, 21 days.	- ↓ blood glucose, atherogenic index, TG, HDL-C, LDL-C, TC, MDA levels, AST, ALP.<br>- ↑ body weight gain, SOD, CAT.	Glucose and lipid metabolism, antioxidant	Not reported
Atta, 2017 [25]	MO leaves methanol extract	Powders of MO leaves were extracted with 95% methanol.	Not reported	CCl4-induced hepatotoxicity in male and female S.D. rats	100, 200, 400 mg/kg BW p.o, 4 weeks.	- ↑ GPx, CAT, GSH, TP, and albumin.<br>- ↓ TC, TG, ALT, AST, MCH, MHCH, RDW, WBC, urea, creatinine.<br>- Improvement of the histopathological picture of the liver.	Antioxidant, lipid metabolism, hepatoprotective	Acute toxicity test:<br>No symptoms of morbidity or mortality.<br>LD50 > 4500 mg/kg BW p.o

Attakpa, 2017 [26]	MO leaves aqueous extract	MO leaves were suspended in distilled water and boiled for 30 min.	Not reported	Spontaneously hypertensive rats.	200, 400, 600 mg/kg BW in food, 8 weeks.	- ↓ blood glucose, TG, LDL, TC levels, BP, arachidonic acid<br>- Inhibition of T cell proliferation, anti-CD3- stimulated T cell blastogenesis, IL-2 secretion<br>- ↑ basal Ca2+, EPA, DHA	Anti-inflammation, Glucose & lipid metabolism, anti-hypertensive	Not reported
Azad, 2017 [27]	Leaves ethanol extract	Powdered leaves of MO were extracted in 80% ethanol.	Not reported	Streptozotocin (STZ) induced diabetes in Long Evan rats.	500 mg/kg BW p.o	- ↓ serum glucose but doesn't affect insulin secretion.<br>- ↓ in the glucose absorption by inhibiting α-amylase<br>- ↑ in gut motility and decrease in starch catabolism.<br>- ↓ amount of glucose in the dialysate.	Glucose metabolism	Not reported
Azevedo, 2018 [28]	MO leaves aqueous extract	Powdered leaves of MO were macerated in distilled water.	Not reported	Streptozotocin- induced diabetes in Wistar rats.	100 mg/kg BW p.o + 200μL of 10% extract topically for 10 days.	- ↓ blood glucose, wound area, TNF-α, IL-1β, and IL-6.<br>- ↑ collagen fibers, fibroblasts, vascular neoformation and macrophages	Anti-inflammation, wound healing	Not reported
Bao, 2020 [29]	Niazirin from MO seeds	MO seeds were boiled twice, followed with extraction using a macroporous resin column and silica gel chromatography. Niarizin was prepared by medium pressure	purity of niazirin: >95%	Saline-induced metabolic syndrome in male db/db mice.	10 and 20 mg/kg/BW p.o for 4 weeks.	- ↓ body weight, water, and food intake,<br>- improved hyperglycemia, insulin resistance, inflammation, carbohydrate and lipid metabolism, non-alcoholic fatty liver.	Anti-inflammation, glucose & lipid metabolism	Not reported

		liquid chromatography from contracted fraction.				- Improved carbohydrate and lipid metabolisms via the AMPK signaling pathway.		
Bitrus, 2018 [30]	MO leaves methanol extract	Powdered MO leaves were extracted using absolute methanol.	10.6%	High cholesterol diet and Carbimazole induced hyperlipidemia and cardiac injury in male albino Wistar rats.	200 mg/kg BW p.o daily for six weeks.	- ↓ TC, LDL, TG, VLDL<br>- ↓ CK-MB, LDH, and AST.<br>- Improvement of histological findings of the heart.	Lipid metabolism, cardio-protective	Not reported
Chen, 2012 [31]	Moringa oleifera leaves hexane extract	Freeze-dried powder of MO leaves were extracted in hexane (1:2 w/v).	Not reported	Monocrotaline-induced pulmonary hypertension in rats	Acute: 1.5, 4.5 and 15.0 mg/kg i.v.<br><br>Chronic: 4.5 mg/kg BW i.p for 7 days	- ↓ pulmonary arterial pressure.<br>- ↑ heart rate<br>- ↑ SOD<br>- ↓ thickening of vessel walls of the histological sections.	Antioxidant, vasculoprotective	Not reported
Cheraghi, 2017 [32]	N,α-L-rhamnopyranosyl vincosamide isolated from MO leaves	Not reported	Not reported	Doxorubicin-induced cardiac toxicity in albino rats.	200, 400, 800, 1000, and 2000 µg/ml i.p every other day for 2 weeks.	- ↑ GSH, SOD.<br>- ↓ MDA, and mRNA levels of β-MHC, ANP, BNP.	Antioxidant, cardioprotective	Not reported
Chin, 2018 [33]	MO leaves aqueous extract	MO leaves were extracted using cold-maceration in distilled water.	Not reported	Streptozotocin and high-fat-diet-induced diabetes in	0.1, 0.5, and 1% film dressing	- ↑ hydroxyproline and COL1α1 levels.<br>- Downregulation of IL-6, TNF-α, and MCP-1 expression at 1%.	Anti-inflammation, wound healing	Acute dermal toxicity: no mortality, no abnormalities on the skin, fur, and

		The extract was formulated with sodium alginate-pectin in film dressing.		male S.D. rats.		- Enhancement of wound healing rate<br>- Promote collagen deposition<br>- Acceleration of wound contraction rate.		behavioral patterns up to 1% dose.<br>No signs of edema, erythema, or any symptoms of toxicity on the skin.
Chumark, 2008 [34]	MO leaves extract	MO leaves were boiled in distilled water for 15 min.	10%	Human LDL;<br><br>High cholesterol diet-induced hyperlipidemia in New Zealand white rabbits	1, 10, 30 and 50 μg/ml.<br><br>0.1 g/kg BW p.o. daily for 12 weeks.	- Delayed the lag-time of conjugated diene formation.<br>- ↓ TBARS formation.<br>- ↓ in TC, LDL, HDL, TG.<br>- Reduced the formation of internal carotid atherosclerotic plaque.	Antioxidant, lipid metabolism, vasculoprotective,	Not reported
Cui, 2019 [35]	MRP-1 isolated from MO roots.	Dried MO roots were boiled in water to extract the polysaccharide MRP-1.	4.6%	RAW264.7 cells treated by LPS	12.5, 25, 50, and 100 μg/ml.	- Inhibition of NO, IL-6, IL-1β and TNF-α production.<br>- Inhibit mRNA expression levels of iNOS and TNF-α.	Anti-inflammation	Lower concentrations (12.5, 25, 50, and 100 μg/ml) had no cytotoxicity while higher concentration (200 μg/ml) showed toxicity.
Das, 2012 [36]	MO leaves ethanol extract.	Powdered MO leaves were extracted using 80% ethanol.	10%	High-fat diet-induced hyperlipidemia in Swiss	150 mg/kg BW p.o for 15 days	- ↑ FRAP, GSH<br>- ↓ MDS, AST, ALT, and ALP.<br>- Prevention of liver damage<br>Prevention of diet-induced	Antioxidant, hepatoprotective	Not reported

				strain male albino mice		obesity and early signs of fatty liver.		
de Yurre, 2020 [37]	Water-Soluble Lectin (WSMoL) isolated from MO seeds	Extraction was done with distilled water, followed with filtration and centrifugation. Afterward, lectin was further saturated, centrifugated, precipitated dialyzed with distilled water.	Not reported	Male C57BL/6 mice	5 mg/kg BW p.o for 21 days	- No change in blood glucose levels, body weight, glucose tolerance or insulin resistance.<br>- No alterations in ECG parameters, cardiac action potential duration, left ventricular and mitochondrial function.	None	No toxicity was observed
Dou Z et al., 2019 [38]	MO leaves extract	a non-digestible fraction of MO leaves was suspended in sterilized basic nutrient growth medium.	Not clearly stated	In vitro gastrointestinal model	5 g/50 mL	- ↓ free radical scavenging activity after gastric and small intestine digestion.<br>- ↓ pH in the colon.<br>- ↑ SCFA and diversity of gut microbiota.	Antioxidant, gut microbiome modulation	Not reported
Edeogu et al., 2019 [39]	MO seed oil	MO seed oil was produced by cold press method with no additional chemicals.	Not reported	Gentamicin-induced nephrotoxicity in male Wistar rats	5 ml/kg BW p.o for 16 days	- ↓ kidney weight, urea, and creatinine.<br>- ↓ MDA IL-6, IL-1b, TNF-α, NO, iNOS, NF-κB, caspase-3.<br>- ↑ GSH, SOD, CAT, and GPx activities.<br>- Histopathological alterations were ameliorated but still showed mild to moderate multifocal	Antioxidant, anti-inflammation, nephroprotective	Not reported

						degeneration and renal tubular epithelial cells necrosis.		
El Rabey, 2017 [40]	MO seed powder	Milled MO seeds were mixed to the 2% cholesterol diet powder.	Not reported	Basal fat-rich diet-induced hypercholesterolemia in male albino rats	50 mg/kg BW for 8 weeks	- ↓ TC, TG, LDL, VLDL, LDH, CK-MB, ALT, AST, ALP, GGT activities.<br>- ↑ HDL,<br>- ↑ water consumption.<br>- Histopathological of the liver showed slight congestion of hepatic sinusoids and nearly restored normal appearance.	Lipid metabolism, hepatoprotective	Not reported
Ezzat SM et al., 2020 [41]	MO leaves ethanol extract	Powdered MO leaves were macerated using 70% ethanol and evaporated to yield dried ethanol extract.	10%	High-fat diet-induced obesity in rats	200 and 400 mg/kg BW p.o for 1 month	- ↓ body weight, adiposity index, glucose, insulin, HOMA-IR, TC, TG, and LDL.<br>- ↑ Increased R-QUICKI, HD.<br>- ↓ leptin and increased adiponectin, omentin, and GLUT-4 levels of the adipose tissue.<br>- Suppressed FAS and HMG-CoA reductase<br>- ↑ mRNA MC4R and PPAR-α.	Lipid metabolism, antiobesity	Not reported
Fahey JW et al., 2019 [42]	MO leaves aqueous tea	Powdered MO leaves were extracted in deionized water.	Not clearly stated	LPS-induced inflammation in RAW264.7 cells.		The doses required to produce a median effect of NO suppression were: moringin, 0.19 µM; cold moringa tea, 0.17 µM and hot moringa tea >100 µM	Anti-inflammation	Not reported
Gao X et al., 2017 [43]	MO leaves aqueous extract	Ultra-micro powder of MO leaves were boiled in ultrapure	31.5%	Healthy male	750 mg/kg BW p.o daily for 4 weeks	- ↓ liver TG content.<br>- Higher levels of bacterial LPS were found in serum.	Anti-inflammation, lipid metabolism, gut microbiome	Not reported

		water, followed by centrifugation. The supernatant was dried in vacuum freeze-dryer.		C57Bl/6 J mice		- Enhanced mRNA expression levels of TNF-α, IL-1β, and IL-6, MCP-1 in the colon.<br>- Upregulation of Reg3g, Pla2g2, Defa in the colon.<br>- Downregulation of Lyz1, Muc2, and occludin.<br>- ↓ ileum mRNA IL-1β, IL-6, increased MCP-1.<br>- Moderate dosage of extract triggered an inflammatory response<br>- Disruption of intestinal homeostasis.<br>- Dominant gut microbial composition was changed.		
Ghasi S et al., 2000 [44]	MO leaves crude extract	Grounded MO leaves were decocted in distilled water for obtaining crude extract.	Not reported	High-fat diet in male Wistar rats	1 mg/g BW p.o once daily for 30 days	- ↓ cholesterol<br>- ↑ albumin<br>- No effect on total protein serum.	Lipid metabolism	Not reported
Gouda AS et al., 2018 [45]	MO extract	Purchased from Egyptian National Research Center (1 g/mL aqueous preparations).	Not reported	Aluminum phosphide-induced acute cardiac toxicity in rats	100 mg /kgBW p.o	- Cardiac muscle showed congestion of intermuscular capillaries with mild disruption and edema of muscular bundles. The extract showed a protective effect.<br>- ↑CAT, GR<br>- ↓ MDA, SOD.	Antioxidant, cardioprotective	Not reported
Gupta R et al., 2012 [46]	Methanol extracts of MO pods (MOMtE)	Dried powdered pods of MO were percolated with 100% methanol.	0.85%	Streptozotocin- induced diabetes in albino rats.	150, 300 mg/ kg BW for 21 days	- ↓ serum glucose, NO, TBARS, MDA.	Antioxidant, glucose metabolism.	Not reported

						- ↑ serum insulin, protein levels, SOD, GSH, CAT.<br>- Reverse the damage of histoarchitectural to the islet cells.		
Huang L et al., 2020 [47]	Isothiocyanates isolated from MO seed	MO peeled seeds were extracted with 30% and 90% methanol for 3 times. It is followed by water-ethyl acetate extraction. The water extract was further fractionated using water and methanol in different concentrations (10%, 30%, 50%, 70%, 90%).	18.5%	3T3-L1 adipocytes	10, 20, 30, 60 µM	Inhibit intracellular lipid accumulation	Lipid metabolism	Not cytotoxic
Huang Q et al., 2020 [48]	MO seeds ethanol extract	MO seed was extracted with water then evaporated, lyophilized and further extraction using 90% ethanol.	4.13%	HepG2-insulin resistance model	10 ng/mL, 100 ng/mL, 1 µg/mL, 10 µg/mL	- ↑ glucose uptake by cells.<br>- Suppressed SRC, PTPN1, caspase-3 expression.	Glucose metabolism, antiapoptotic	Not reported
Irfan HM et al., 2016 [49]	MO leaves ethanol extract	Powdered MO leaves were extracted with 95% ethanol by maceration.	Not reported	Streptozotocin- induced diabetes male S.D. rats	125, 250, 500, 1000 mg/kg BW p.o for 14 days	↓ fasting blood glucose concentration, body weight, cholesterol, TG.	Glucose and lipid homeostasis	Not reported

Jaiswal D et al., 2009 [50]	MO leaves aqueous extract	Crushed MO leaves were extracted hot distilled water.	11.7%	Streptozotocin- induced diabetes male albino Wistar rats	100, 200, 300 mg/kg BW p.o for 21 days	- ↓ fasting blood glucose, postprandial glucose, urine glucose, and protein.<br>- ↑ Hb and total protein.	Glucose metabolism	Acute toxicity test: not toxic up to 15 x effective dose. Normal behavior and no death.
Jaiswal D et al., 2013 [51]	MO leaves aqueous extract	Fresh leaves macerated in distilled water.	10.5%	Streptozotocin- induced diabetes male albino Wistar rats	200 mg/kg BW p.o daily for 21 days	- ↑ SOD, CAT, GST.<br>- ↓ MDA	Antioxidant	Not reported
Jaja-Chimedza A et al, 2017 [52]	Isothiocyanate-enriched MO seed extract	MO seed extract was prepared by incubating ground seeds in water, then ethanol, followed by filtration, drying and purification.	12.6 - 13.2%	RAW 264.7 cells<br><br>Carrageenan-induced paw edema in male S.D. rats	0.05, 0.1, 0.5, 1, 5 and 10 µM<br><br>250 and 500 mg/kg BW	- ↓ NO production, iNOS, IL-1β and IL-6 expression.<br>- Upregulation of all Nrf2 target genes (NQO1, HO1, GSTP1).<br>- Inhibition of paw edema.	Anti-inflammation	No significant effect on cell viability.
Joung H et al, 2017 [53]	MO fermented extract	Powder of MO leaves was mixed in distilled water in three different mixtures. Those were inoculated with 2.5% culture media of each strain and incubated 37°C for 24 h. Samples were centrifuge at 4 °C, 12000 rpm, 15 min.	Not reported	High-fat diet-induced obesity in male C57BL/6J mice	250 mg/kg BW p.o daily for 10 weeks	- Improved glucose tolerance test.<br>- ↓ liver weight, hepatic lipid accumulation.<br>- Downregulation of lipogenic genes (ACC, FAS, C/ENPα, SREBP1c, LPL), lipid oxidative genes (CD36, ACOX1, CPT1 (NFM), HSL), and oxidative stress genes (UCP2 and UCP3).<br>- Upregulation of lipolysis genes (ATGL, HSL).<br>- Reduced expression of genes<br>- related to ER stress (BiP, PDI, and CHOP).	Glucose metabolism, antioxidant, anti-inflammation	Not reported

						- ↓ mRNA proinflammatory cytokines (TNFα, IL-6, and IL-12).<br>- Unchanged Akt phosphorylation level.		
Khalil SR et al., 2020 [54]	MO leaves ethanol extract	Powdered MO leaves was macerated in absolute ethanol	Not reported	Tilmicosin-induced cardiac injury in male S.D. rats	400 and 800 mg/kg BW p.o	- ↓ mortality rate.<br>- ↓ CK-MB, CPK, troponin levels.<br>- ↓ MDA, PC, and 8-OHdG levels.<br>- ↑ CAT, SOD, GSH, TAC.<br>- Up-regulation of Bcl-2<br>- Downregulation of Bax, caspase-3, Apaf-1, AIF, P53.<br>- Heart sections revealed partial restoration.	Antioxidant, antiapoptotic, cardioprotective	Not reported
Kumar Gupta S et al., 2013 [55]	MO leaves aqueous extract	Dried leaves were extracted using water at 60-70°C.	Not reported	Streptozotocin- induced diabetes male albino Wistar rats	100 mg/kg BW p.o for 24 weeks	- ↓ blood glucose levels, HbA1c.<br>- ↓ retinae dilated vessels and<br>- Improvement of the thickened basement membrane.<br>- ↓ clinical grading of fluorescein angiograms.<br>- ↓ retinae TNF-α, IL-1β, VEGF, PKC-β.<br>- ↑ retinae GSH, SOD, CAT	Antioxidant, anti-inflammation, Vasculoprotective	Not reported
Li C et al, 2020 [56]	Novel polysaccharides from MO leaves	(1) Hot water extraction followed by ethanol precipitation (crude MOPL)	Crude MOPL: 6.84%; further purification from	RAW 246.7 cells	31.3–500 µg/mL	- Stimulation of pinocytosis, ROS, NO, IL-6 and TNF-α<br>- ↑ mRNA of NO, IL-6 and TNF-α.	Immunomodulation	Cytotoxicity test:<br>Doses 31.3–500 µg/mL promoted cell proliferation.

		(2) Further purification using DEAE-Sepharose fast-flow ion-exchange	MOPL: 15.4%					Not toxic to cells.
Li YJ et al, 2020 [57]	MO seed	Not reported	Not reported	Myocardial infarction (MI) male C57/BL6 mice	600 mg or 900 mg in food, daily for 2 weeks pre- and post-MI surgery	- ↑ survival rate.<br>- ↑ LVEF and LVFS) in ECG analysis.<br>- ↓ heart volume, infarct areas, the volume of infarcted heart, and fibrotic scarring.<br>- The thicker anterior wall of the left ventricle chamber.<br>- Prevent cardiac remodeling.<br>- ↓ TUNEL positive cells, Bax, cytochrome C<br>- ↑ Bcl-2, which indicates reduced myocardial apoptosis.<br>- ↓ gp91phox, and iNOS.	Antioxidant, antiapoptotic, cardio-protective	Not reported
Liao PC et al, 2018 [58]	β-Sitosterol isolated from MO woody stems	Woody stems of MO were extracted using ethanol followed with partitioned in *n*-hexane/water (1/1 v/v).<br>The *n*-hexane fraction was applied to a silica gel column and then eluted with acetone/n-hexane (1:1 v/v), followed with purification in	5.9%	HaCaT and J774A.1 cells	7.5−60 μM	- ↓ IL-1β, IL-6, IL-8, TNF-α secretion.<br>- Inhibition of NLRP3, expression, ROS production, caspase-1 activation.<br>- Partially inhibited NF-κB in macrophages.	Anti-inflammation	Cytotoxicity test: no toxicity up to 60 μM

		HPLC to obtain stigmasterol and β-Sitosterol						
López M et al, 2018 [59]	MO leaf powder	Not reported	Not reported	High-fat and fructose diet-induced metabolic syndrome male Wistar rats	Preventive: 700 mg/kg BW p.o daily for 3 weeks before induction<br><br>Treatment: 700 mg/kg BW p.o daily for 3 weeks after induction	- ↓ fasting glucose levels.<br>- There are no significant differences in ITT, OGTT, TC, TG, SBP, DBP, or abdominal circumference.<br>- ↓ glucose tolerance, TG, and abdominal circumference.<br>- No significant differences in the ITT.	Glucose and lipid metabolism	Not reported
Luetragoon T et al., 2020 [60]	MO ethyl acetate extract and fractions	Dried MO powder was extracted by hexane and ethyl acetate (EtOAc) individually.<br>Crude EtOAc were fractionated in column chromatography.	2.2% (hexane); 6.4% (ethanol)	Human MDM	Ethyl acetate extract: 56.98 µg/mL<br>fraction 6: 144.66 µg/mL<br>fraction 12: 162.08 µg/mL	- Downregulation of mRNA IL1, IL-6, TNF-α, PTGS2, NF-κB (P50), and RelA.<br>- Inhibition of IL-6 and TNF-α production.<br>- Inhibition of NF-κB (p65) translocation into the nucleus leads to the reduction of p65, phospho-IκB-α, and COX-2 proteins levels.	Anti-inflammation	Cytotoxicity test:<br>LC50 of ethyl acetate extract, fraction 6, fraction 12 were 521.19, 693.42, 1119.43 µg/mL, respectively.
Mabrouki L et al., 2020 [61]	methanol extract of MO leaves	Fine powdered MO leaves were extracted with methanol.	Not clearly stated	in vivo: Rats ((high-fat diet-induced obesity)	200 mg/kg/bw and 400 mg/kg/bw for 12 weeks	- ↓ body weight gain<br>- ↓TC, ↓ TG, ↓ HDL-C<br>- ↑ CK-MB<br>- ↑ CAT, GPx, SOD<br>- ↓ MDA	Lipid metabolism, antioxidant	not reported

Madkhali HA et al., 2019 [62]	methanol extract of MO leaves (MEMO)	Powdered MO leaves was extracted with methanol using a Soxhlet apparatus	Not reported	in vivo: Rats (high-fat diet-induced dyslipidemia and vascular endothelium dysfunction)	200 and 400 mg/kg/day for 3 weeks	- ↓ liver weight<br>- ↓ BMI, ↓ waist, ↓ Lee-index<br>- ↓TC, ↓TG, ↓VLDL, ↓LDL, ↑HDL-C<br>- reversing endothelial dysfunction (↓ endothelium relaxation)<br>- improvement and photomicrographs architecture of aorta	Lipid metabolism, vasculoprotective	not reported
Mapfumo M et al., 2019 [63]	Crude hydroethanolic MO seed extract	Dried and dehulled MO seeds were grounded. It is extracted with 70% ethanol to obtain seed extract.	Not reported	in vivo: Rats (high fructose diet for induced metabolic syndrome)	50 and 500 mg/kg body mass for 12 weeks	- ↓ glucose tolerance (more pronounced in male than female rats)<br>- ↓ fasting glucose, insulin, and HOMA-IR (more favorable in female than male rats)<br>- ↓ TG<br>- no change in total cholesterol	Glucose and lipid metabolism	not reported
Mehta K et al, 2003 [64]	MO fruit powder	The outer skin, pulp, and seed of MO fruits were collected and dried at 60 °C separately and then sifted.	3 - 7%	in vivo: Rabbits (hypercholesterolemia diet)	200 mg/kg p.o for 120 days in banana pulp	- ↓ TC, ↓ phospholipid, ↓ TG, ↓ VLDL, ↓LDL-C, and ↑ HDL ratio (HDL/HDL-TC)<br>- ↓HDL-C<br>- ↓ lipid profile in liver, heart, and aorta, but not in heart	Lipid metabolism	not reported
Muhammad AA et al., 2016 [65]	MO aqueous fraction	The dried and powdered leaves were macerated with 80% methanol, then was separated in hexane and dichloromethane, as well as butanol and ethyl-acetate. and water:methanol (1:3 v/v).	Not reported	*in vitro*: Pathogenic bacteria consisting of *P. aeruginosa* strain PAO1, methicillin-resistant *S.*	0.5%, 1%, and 2% w/w in ointment formulation	*in vitro*<br>- inhibited growth *S. aureus*: 3.125 µg/mL and P. *aeruginosa*: 6.25 µg/mL against *E. coli*<br>- The MIC for tetracycline standard antibiotic: 3.125 µg/mL against *E. coli* and 6.25 µg/mL against *S. aureus* and *P. aeruginosa*.<br><br>*in vivo*:<br>↓ wound size	Antiinflammation, wound healing, antibacterial	not reported

				aureus, and *E. coli*<br><br>*in vivo*:<br>Rats (streptozotocin and nicotinamide-induced diabetic, followed by wound excision)		↑ wound contraction<br>↑ tissue regeneration<br>↓ inflammatory mediators: TNF-α, IL-1β, IL-6,iNOS, and COX-2<br>↑ VEGF		
Murillo G et al., 2017 [66]	MO leaves powder	Not reported	Not reported	In vivo: guinea pigs (hypercholesterolemic diet with 0.25% cholesterol)	3 or 4.5 g/day a daily intake for 6 weeks	- no changes in plasma lipids, glucose, or insulin<br>- ↑ the activity of lecithin cholesterol acyltransferase	Lipid metabolism	not reported
Nafiu AO, 2019 [67]	Ethanolic extract of MO seeds	MO seeds were extracted in ethanol for 24 h.	Not reported	in vivo: Rats (gentamicin-induced nephrotoxicity)	100, 200 and 400 mg/kg/day p.o for 28 days	- ↓ urine volume<br>- ↑ body weight<br>- ↓ urea in urine<br>- ↓ creatinine plasma and ↑ creatinine urine<br>- ↑ creatinine clearance<br>- restoration electrolytes<br>- ↑ SOD and ↓ MDA<br>- improvement in the histoarchitecture of the kidney with mild distortion, few abortive glomeruli, and mild vascular congestion (400 mg/kg treatment seems to have fared better than others)	Antioxidant, nephroprotective	not reported

Nandave et al, 2007 [68]	Lyophilized hydroalcoholic extract of *Moringa oleifera*	MO leaves were macerated with (methanol: water, 50:50 v/v) and evaporated to yield the extract.	34.2%	in vivo: Rats (isoproterenol-induced) cardiotoxicity model	200 mg/kgBW for 1 months	- improvement hemodynamic parameters<br>- ↑ SOD, CAT, GSH-Px, LDH, and CK-MB<br>- prevention of the deleterious histopathological and ultrastructural perturbations	Antioxidant, cardioprotective	not reported
Ndong et al, 2007 [69]	MO leaves powder	Not reported	Not reported	in vivo: Rats (iron-deficient diet)	0,5-1% MO in a diet for 4 weeks	- ↓ Hb<br>- ↑ liver weight<br>- ↓ liver iron<br>- ↓ liver copper<br>- ↓ TC, TG, and phospholipids	Lipid metabolism	not reported
Ofem, O.E et al., 2015 [70]	Aqueous leaves of MO	Grounded MO leaves were extracted with distilled water at 100 °C for 9 hr using Soxhlet apparatus.	31%	in vivo: Rats (high salt diet)	600 mg /kgBW for 6 weeks	Resolved hematology parameter: ↑WBC, RBC, platelet counts, PCV, lymphocytes, P-LCR, MPV, and PDW	Hematology	not reported
Olayaki et al, 2015 [71]	Methanolic extracts of MO (MOLE)	Powdered MO leaves were soaked with methanol using a cold-extraction method.	Not reported	in vivo: Rats (alloxan-induced diabetes)	300 and 600 mg/kg body weight p.o for 6 weeks	- ↓ blood glucose<br>- ↑ glucose tolerance<br>- ↑ serum insulin<br>- ↓ serum cholesterol, TG, LDL-C, ↑ HDL<br>- ↑ glycogen synthase activities and glycogen contents.	Glucose and lipid metabolism	not reported
Oldoni TLC et al., 2021 [72]	Hydroalcoholic subfraction extract leaves of MO	Grounded MO leaves were extracted with ethanol water (80:20) for 48 hr at room temperature (HE). The HE was fractioned by solid-	Not reported	*in vitro*:<br>● Folin-Ciocalteu assay<br>● Ferric reducing antioxidant	in vitro: 25 - 1000 µg/mL hydroalcoholic extract<br><br>in vivo: 500 mg/kg	Fr-EtOAc :<br>- ↓ FRAP, DPPH and ABTS, and ORAC assay<br>- isoquercitrin, astragalin and 3-O-caffeoylquinic acid were obtained.<br>In vivo:<br>- ↓ blood glucose	Antioxidant	no observed cytotoxicity effect (concentration up to 1000 mg/mL)

		liquid extraction using hexane, dichloromethane , ethyl acetate , acetone, ethanol and ethanol:water (50:50, v/v). The active Fr-EtOAc was purified in an open Column Chromatography and followed by TLC to obtain subfractions.		power (FRAP) assay<br>● DPPH assay<br>● ABTS assay<br>● cell viability using PBMCs<br><br>*in vivo*:<br>Rats (streptozotocin-induced)	hydroalcoholic extract	- ↑ CAT activity in liver and kidney<br>- ↓ GST in liver and kidney<br>- ↓ non-protein thiol groups and thiobarbituric acid reactive substances in liver and kidney		
Omodanisi EI et al., 2017 [73]	Methanolic extract of MO leaves	Not clearly stated	Not reported	in vivo:<br>Rats (streptozotocin-induced)	250 mg/kg BW p.o for 6 weeks	- ↑ renal weight<br>- ↓ plasma glucose<br>- ↓ MDA, ↑ SOD, ↑CAT, and ↑ GSH in renal<br>- ↓ TNF-α and ↓ IL-6<br>- protective effect on histological examination	Antioxidant	not reported
Ouédraogo M et al., 2011 [74]	Aqueous-ethanolic extract of MO leaves	The powdered MO leaves were extracted with ethanol/water (80/20) for 48 h at room temperature.	4.8%	in vivo:<br>Rabbit (nephrotoxicity induced by gentamicin)	150 and 300 mg/kgBW	- ↓ serum urea and creatinine<br>- ↓ lipid peroxidation<br>- reparative tendencies from histological examination	Antioxidant, lipid metabolism, nephroprotective	no toxicity signs were observed
Panda S, 2013 [75]	N,a-L-rhamnopyranosyl vincosamid	70% ethanol of powdered leaves extract	Not reported	in vivo:<br>Rats (isoproterenol-induced	40 mg/kgBW for 7 days	- ↓ serum LDH, CK-MB, troponin T, and glutamate pyruvate transaminase<br>- ↓ cardiac Troponin T and MDA	Antioxidant, cardioprotective	not reported

	e (VR) isolated from MO leaves			cardiotoxicity model)		- ↓ myocardial necrosis		
Panda, 2015 [76]	MO poly-phenolic fraction (MOPF)	Powdered MO leaves was extracted with 70% ethanol. The crude extract was fractionated using n-hexane, dichloromethane, and n-butanol.	Crude extract: 20.1%; n-hexane: 1.1%, dichloromethane 0.9%; n- butanol: 1.9%	in vivo: Rats (isoproterenol-induced cardiotoxicity model)	50, 100 and 150 mg/kg/day for 28 days	- ↑ SOD, ↑ Catalase, ↑ GSH-Px, ↑ GSH (MOPF100)<br>- ↓ cardiac infarcted area (MOPF100)<br>- ↓LDH, CK-MB, and cardiac Troponin T (MOPF100)	Antioxidant, cardioprotetive	not reported
Paula PC et al., 2017 [77]	Isolated protein from MO leaves: Mo-LPI	Powdered MO leaves was extracted in Tris-HCl buffer containing NaCl, polyvinylpolypyrrolidone (PVPP), phenyl-methyl-sulfonyl fluoride (PMSF) and ethylenediaminetetraacetic acid (EDTA), (1:5, w/v), under agitation at 4 ◦C for 30 min. The crude extract was precipitated at	5%	in vivo: Mice (alloxan-induced diabetes)	500 mg/kgBW, single-dose intraperitoneal (i.p.)	- ↓ blood glucose<br>- ↓ MDA and ↑ catalase activity	Antioxidant glucose metabolism	Mo-LPI (2500 mg/kgBW) did not cause acute toxicity to mice.

		90% saturation with ammonium sulphate.						
Rajanandh, M.G, et al., 2012 [78]	Hydroalcoholic extract of Moringa oleifera (HEMO)	Powdered MO leaves were extracted with ethanol using cold maceration methods.	0.09% of Beta-sitosterol	in vivo: Rats (atherogenic diet for inducing hyperlipidemia)	200 mg/kg p.o. for 28 days	- ↓ body weight<br>- ↓ TC, TG, LDL-C, VLDL, and ↑ HDL-C<br>- ↑ SOD, ↑ CAT, and ↓MDA<br>- ↓ TNF-α and ↓ IL-1α	Antioxidant anti-inflammatory, lipid metabolism	Not reported
Randriamboavonjy JI et al, 2016 [79]	MO seed powder	Not reported	Not reported	*in vivo*: Wistar Rats induced by spontaneous hypertensive	750 mg/d each rat in their food for 8 weeks	- did not modify BP<br>- ↓ nocturnal heart rate<br>- ↓ isovolumetric relaxation time and deceleration time of the E wave<br>- ↑ ejection volume and cardiac output<br>- ↓Left ventricular anterior wall thickness, interseptal thickness on diastole, and relative wall thickness<br>- ↓fibrosis in the left ventricle<br>- ↑ PPAR-α and δ<br>- ↑ plasmatic prostacyclins	Cardio-protective	Not reported
Randriamboavonjy JI et al, 2017 [80]	MO seed powder	Not reported	Not reported	in vivo: Wistar Rats induced by spontaneous hypertensive	750 mg/day/rat in food for 20 weeks	- ↓ free 8-isoprostane circulating level, vascular p22phox and p47phox expressions<br>- ↑ SOD2<br>- ↓ circulating nitrites and CRP<br>- ↓ iNOS and NF-κB<br>- ↑ endothelium-dependent carbachol-induced relaxation	Antioxidant, anti-inflammatory	Not reported

Reddy VP et al., 2017 [81]	MO leaves polyphenols (MOP)	Dried MO leaves were extracted with 80% methanol.	Not reported	in vivo: Wistar Rats (high fat-cholesterol diet for 45 days)	100 and 200mg/kg p.o for 8 weeks	- ↓ TC, LDL-C<br>- ↓ HMG-CoA<br>- ↑ fecal bile acid	Lipid metabolism	not reported
Saka WA et al, 2020 [82]	MO seed oil	Pounded MO seeds were extracted with 97% ethanol using Soxhlet apparatus at 78 °C.	Not reported	in vivo: rats induced by DDVP via inhalation for 15 min daily for 28 days	300 mg/kg p.o for 28 days.	- ↓ lactate dehydrogenase, creatinine kinase, and troponin<br>- ↓ MDA<br>- ↑ SOD and GSH-Px	Antioxidant	Not reported
Sangkitikomol W et al, 2014 [83]	Methanol MO extract (MOE)	Powdered MO leaves were extracted with 80% methanol in an ultrasonic bath for 60 min at 40 °C and stored in the dark followed by centrifugation.	20%	in vitro: HepG2 cell line	Cell viability MOE (0-3000 mg/L)<br>Treatment: 0,200,400, and 600 mg/L	- ↓ oxidative stress in a dose-dependent manner (MOE >100 mg/L, 24 h)<br>- ↓ HMG-CoA-Reductase, PPARα1, and PPARγ genes expression	Antioxidant	MOE induced cytotoxicity in high dose (2000-3000 mg/L)
Shaikh S et al., 2020 [84]	MO root extract	Dried powder of roots MO was extracted serially in methanol followed by boiled in a water bath to evaporate methanol.	Not reported	in vitro<br>- DPPH assay<br>- Pancreatic amylase assay	100 to 1000 µg/mL with an interval of 100µg/mL	- MO IC50 DPPH assay: 480.64 µg/mL<br>- MO IC50 pancreatic amylase assay: 728.97µg/mL	Antioxidant, glucose metabolism	not reported
Sholapur HN et al., 2013 [85]	Alcoholic and petroleum ether	Powdered bark was macerated in 80% ethanol and petroleum ether.	Not reported	in vivo: Rats were induced by dexamethas	Acute and chronic toxicity studies:	Alcoholic Extract 125 and 250 mg/kg:<br>- ↓ TG and oral glucose intolerance but not fasting hyperglycemia	Lipid metabolism	no toxicity was observed

	extracts of MO bark			one for 11 days	- 2 doses of alc. extract (125 and 250 mg/kg)<br>- 2 doses petroleum ether extracts (30 and 60 mg/kg)<br>- single dose each of alc. extract (250 mg/kg)<br>- single dose of petroleum ether extract (60 mg/kg)	Petroleum ether extract 30 and 60 mg/kg:<br>- PEE30 had no effects<br>- PEE 60 ↓TG		
Sinha et al, 2011 [86]	Aqueous ethanolic MO leaves extract (MOLE)	Powdered MO leaves were extracted with 50% methanol	Not reported	in vivo: Swiss albino mice using single dose of 5 Gy of 60Co c-irradiation	MOLE p.o 300 mg/kg of body weight for 15 days	- ↓ translocation of NF-kB from the cytoplasm to the nucleus<br>- ↓ lipid peroxidation<br>- ↑SOD, CAT, GSH, and FRAP	Antioxidant	not reported
Soliman MM et al., 2020 [87]	MO leaf extract (MOLE)	MO leaves soaked in a hydro-alcoholic solution and shaken at room temperature for 48 h.	Not reported	In vivo: mice induced by Methotrexate on day 7	MOLE p.o 300 mg/kgBW, 12 days.	- ↓ ALT, ALP, urea, and creatinine in serum<br>- ↑ albumin and total protein<br>- ↓ MDA, ↑ SOD and ↑catalase in serum<br>- ↓ MDA, ↑ SOD, and ↑GSH in liver and kidney<br>- Liver: ↓ BAX, ↓ TIMP, ↑XIAP mRNA expression level	Antioxidant, antiapoptotic	not reported

						- Renal: Nrf2 unchanged, ↑HO-1, ↓ NF-kB mRNA expression level<br>- Restored liver histopathology architecture<br>- Restored renal histological architecture<br>- ↓ caspase-9 in liver and renal<br>- ↑ Bcl2 in liver and renal		
Sun C et al, 2019 [88]	1-O-(4-hydroxymethylphenyl)-α-L-rhamnopyranoside (MPG) from MO seeds	Seed powder were de-oiled with petroleum ether and ddH2O at 95°C. n-Butanol was used for further fractionation, followed with sub-fractionation with MOE-BuOH and chromatography.	18.1%	*in vitro*: LO2 cells induced by CCl4<br><br>*In vivo*: ICR mice induced by single dose CCl4 on 8th days treatment	50, 100, 150 mg/kg BW p.o for 7 days	*in vitro*:<br>- ↑ cell viability<br>- ↑ intracellular SOD activities<br>- ↓ LDH<br>- leakage in CCl4-treated cells.<br>*in vivo*:<br>- ↓ abnormal enlargement liver weight<br>- ↓ ALT, ALP, AKP in serum<br>- improve histopathological analysis<br>- enzymatic antioxidant activities (↑SOD, ↑CAT, and ↑GSH-Px)<br>- non-enzymatic antioxidant activities (↑T-AOC and ↑GSH)<br>- ↓ hepatic MDA level<br>- ↓ hepatic ROS production<br>- ↓ TNF-α, IL-1β, and MCP-1 levels in liver<br>- ↑ IL-10 in the systemic circulation<br>- ↓ apoptotic hepatic cells	Antioxidant, anti-inflammatory, antiapoptotic, hepatoprotective	- no notable cytotoxic effects at 24 h, 48 h, and 72 h (5–100 µg/mL)<br>- first phase acute oral toxicity in mice: up to 1000 mg/kg→ no adverse effects and death<br>- second phase oral toxicity in mice: up to 2000 mg/kg single administration → no adverse effects and death

Tan WS et al, 2015 [89]	80% hydroethanolic extract of MO flower	MO flower powder was macerated in hydroethanolic solvent under a rotary shaker at room temperature.	Not reported	*in vitro*: The murine macrophage cell line, RAW 264.7,	MTT assay: 15.625 - 1000 µg/mL<br>*in vitro*: 100 µg/mL and 200 µg/mL	• ↓ secretion and expression of NO,PGE2, IL-6, IL-1β, TNF-α, NF-κB, iNOS, and COX-2.<br>• ↑ IL-10 and IκB-α<br>• dose-dependent manner (100 µg/mL and 200 µg/mL).	Antioxidant, anti-inflammatory	Not exhibited any toxicity to macrophages at concentrations ranging from 15.625 to under 250 µg/mL.
Tang Y et al, 2017 [90]	Aqueous and ethanolic extract MO leaves, stems, and seeds	Leaves, stems, and seeds of MO were boiled separately distilled water and with 70% ethanol.	Not reported	*in vivo*: db/db mice	MO leaves ethanolic extract 150 mg/kg/day for 5 weeks (gastric intubation)	DPPH activity:<br>ethanolic leaf extract the strongest hydroxyl radical scavenging activity<br>*In vivo*:<br>- ↓ fasting plasma glucose, triglyceride, and LDL cholesterol<br>- ↑ insulin levels<br>- ↓ histopathological damage, TNF-α, IL-1β, IL-6, cyclooxygenase-2, and iNOS in renal tissue	Glucose metabolism, anti-inflammatory, nephroprotective	not reported
Tian et al, 2021 [91]	MO polysaccharides (MOP)	MO powder was macerated in boiled distilled water and centrifuged. Afterwards, the supernatant was collected and concentrated.<br>The Sevag method was used to remove the protein impurities.	Purity of MOP: 83.42%	in vivo: male SPF C57BL/6 mice	MOP p.o: 0, 20, 40 and 60 mg/kg bw for 30 days	Serum:<br>- ↓ glucose, total cholesterol, & MDA<br>- Improved SOD and catalase in serum<br>Small intestine<br>- Improved the villi length and crypt depth in both ileum and jejunum<br>- ↑ the ratio of villi length to crypt depth in jejunum<br>- ↑ beneficial bacteria<br>- ↓ harmful bacteria in the caecum<br>- MOP regulated 114 metabolites enriched in the pathway related to the synthesis and metabolism of micromolecules.	Glucose metabolism, antioxidant, gut microbiome	not reported

Umar et al, 2018 [92]	Hexane, ethyl acetate, and methanol extracts of MO root	MO roots were extracted with hexane, ethyl acetate, and methanol separately in Soxhlet apparatus at 50°C.	Not reported	in vivo: male *Rattus norvegicus*, albino variety induced by alloxan	Powder root extract: 15g/kg BW/day p.o for 4 weeks. methanolic extract of MO roots (1 g/kg BW/day, i.p. injection for 4 weeks.	- ↓ fasting blood glucose levels<br>- ↓ insulin resistance<br>- ↑ SOD, CAT, GPx	Glucose metabolism, antioxidant	- LD50 was above 5000 mg/kg<br>- no death observed
Vargas-Tineo et al., 2020 [93]	Aqueous extract of MO leaves	Water extraction at 90 C	Not reported	in vivo: male *Rattus norvegicus*, albino variety induced by alloxan	200 mg/kg po	↓ glycemia	Glucose metabolism	Not reported
Velaga MK et al., 2014 [94]	M. oleifera seed powder	Powder MO seeds were suspended in distilled water.	Not reported	in vivo: Male Wistar rats induced by 2000 ppm of lead acetate for 2 weeks	500 mg/kg p.o on 7 days after lead acetate for 1 week	Brain, liver, and kidney<br>- ↓ reactive oxygen species (ROS)<br>- ↓ lipid peroxidation products (LPP)<br>- ↓ total protein carbonyl content (TPCC)<br>- ↓ metal content<br>Blood<br>- ↑ delta-aminolevulinic acid dehydratase (ALAD) activity<br>- ↑ RBC, WBC, hemoglobin, and hematocrit	Antioxidant	Not reported
Vera-Nuñez N et al., 2021 [95]	Lectin on MO seeds: WSMoL	Distilled water and ammonium sulfate were used to extract MO seeds, followed with centrifugation. The precipitate was	Not reported	in vivo: adult C57BL/6 mice by combining a high-fat diet	WSMoL: 5 mg/kg bodyweight for 21 days	- ↓ fasting blood glucose levels<br>- ↓ insulin resistance<br>- improve cardiac LV ejection fraction	Glucose metabolism, cardio-protective	no adverse effects on body weights were observed

		resuspended and dialyzed. Using a chitin column, the dialyzed fraction is loaded and the WSMoL is eluted with an acid solution of sodium acetate.<br>Dialyzed against distilled water, the isolated lectin is characterized.		and low doses<br>of STZ (6 wk)				
Villarruel -López A et al, 2018 [96]	Powdered leaves extract MO	Dried leaves from 40°C (24h) were pounded.	Not reported	in vivo-toxicity: Balb-C56 male mice<br><br>in vivo-diabetic rats: Alloxan on S.D. rats	Acute toxicity: 100, 200, 500 mg/kg of Moringa oleifera for 7 days observed<br><br>Diabetic-rats: Moringa oleifera was 50 mg/day, and the glibenclamide dose was 600 µg/kg/day for 8 weeks	- hypoglycemic effect<br>- no change in enumeration of lactic acid bacteria	Glucose metabolism, gut Microbiome	- no lethal dose was determined<br>- no adverse effects were observed<br>- no genotoxicity was observed
Wang F et al, 2019 [97]	Niazirin from aqueous MO seed	Not reported	Not reported	in vitro: VSMCs (vascular	in vitro: 5 and 25 uM	- DPPH<br>- ↓ radical scavenging activity<br>- in vitro and in vivo<br>- ↓ the proliferation of high glucose-induced VSMCs	Antioxidant	not observed

				smooth muscle cells)<br><br>in vivo: ICR mice (high-fat diet for 8 wks+ inject STZ 40 mg/kg)	in vivo: 10 and 40 mg/kg/day for 2 weks	- ↓ ROS and MDA<br>- ↑T-AOC, SOD, GPx levels<br>- eliminate the high glucose-induced PKCζ activation, indicated by Thr410 phosphorylation and inhibition of the Nox4 protein expression		
Wang F et al, 2019 [98]	Polysaccharide from MO leaf: MOs-2-a	Chopped MO leaves were extracted using by deionized water and loaded into a macro-porous resin column. Ethanol was added, centrifuged and dried to obtain crude polysaccharide.	Not reported	in vivo: ICR mice	(50 mg/kg for MOs-2-a-H group and 10 mg/kg for MOs-2-a-L group)	- ↓ TNF-α, diamine oxidase (DAO), and d-lac levels in the intestine<br>- improve the integrity of intestinal tissue in MOs-2-a group (villus height & mucosal thickness)<br>- ↑ the activity of amylase; Lipase; Alkaline phosphatase; Trypsin.<br>- ↓ the nitrogen content of feces<br>- keep the pH at a faintly acidic level<br>- restored gut microbiota composition<br>- ↑ the bacteria for anti-obesity effects, SCFA, and lactic acid production.	Antioxidant, gut microbiome	28 days observation:<br>- BW higher than in mice fed standard<br>- increase the viscera index of the spleen and thymus
Wang F et al, 2017 [99]	The macroporous resin adsorption extract of MO seeds:	MO seeds were extracted two times with boiling water and passed over a macro-porous resin column to gain a crude extract. It was loaded into Medium Pressure	Not reported	in vitro: HepG2 cell<br><br>in vivo: STZ-induced mice.	in vitro: 10, 20, 40, 80, 160, dan 320 ug/mL<br><br>in vivo: dose of 20 mg/kg/day,	Compound 1, 4, and 5:<br>- promote the glucose consumption of insulin resistance cells<br>- reduce blood glucose levels of STZ-induced mice.	Glucose metabolism	

		Preparative Liquid Chromatography with an ODS column to obtained niazirin.						
Waterman C et al., 2015 [100]	Moringa isothiocyanates (MICs)	Fresh moringa leaves were blended in 25°C water at 25°C for 30 min, centrifuged, filtered, and lyophilized.	3	in vivo: C57BL/6L mice fed a very high-fat diet (VHFD)<br><br>in vitro: H4IIE rat hepatoma cells	66 mg/kg/d of MICs for 12 weeks	in vivo:<br>- ↑ glucose tolerance and insulin signaling (IRS-1, p-IRS-1 in the liver)<br>- ↑ p-IRS1, IRS1, IRS2, IR β, and GLUT4 in the muscle<br>- did not develop fatty liver disease<br>- ↓ plasma insulin, leptin, resistin, cholesterol, IL-1β, TNFα,<br>- ↓ hepatic G6P expression.<br>in vitro:<br>Inhibition of gluconeogenesis and G6P expression.	Glucose metabolism, anti-inflammatory	without any other observable side effect (12 weeks)
Yang Y et al, 2020 [101]	Bioactive compound :O-Ethyl-4-[(α-l-rhamnosyloxy) benzyl] carbamate	Not clearly stated	Not reported	DPP-IV assay		IC_{50} = 798 nM to inhibit DPP-IV	Glucose metabolism	not reported
Yassa HD et al., 2014 [102]	Aqueous extract of MO leaves	Dried and powdered leaves were boiled in water for 15 min.	Not reported	in vivo: 40 S.D male albino rats STZ-induced 60 mg/kg BW in 0.1 mol/L citrate buffer (pH 4.5) i.p	200 mg/kg aqueous extract of MO leaves (8 wk)	- ↓ glutathione and lipid peroxidation product and MDA, in pancreatic tissue.<br>- Amelioration of the altered FPG (from 380% to 145%), reduced glutathione (from 22% to 73%) and MDA (from 385% to 186%) vs control.	Antioxidant	not reported

				after overnight fasting		- Marked reversed histopathological damage of islet cells.<br>- ↑ areas of positive purple modified Gomori stained-cells (from 60% to 91%) morphometrically<br>- ↓ percentage of collagen fibers (from 199% to 120%) vs. control.		

ALP: alkaline phosphatase, ALT: alanine aminotransferase, ANP: atrial natriuretic peptide, AST: aspartate aminotransferase, AOPP: advanced oxidation protein products, BiP: Binding immunoglobulin protein, BUN: Blood urea nitrogen, BNP: B type natriuretic peptide, BP: blood pressure, BW: body weight, CAT: catalase, CD: conjugated dienes, CHOP: CCAAT-enhancer binding protein homologous protein, CK-MB: Creatine kinase-MB, COL1α1: collagen type I alpha I, COX-2: cyclooxygenase-2, Cr: Creatinine, CRP: C-reactive protein, DDVP: dichlorvos, DPPH: 2,2-diphenyl-1-picrylhydrazyl, ECG: electrocardiogram, FAS: fatty acid synthase, FPG: fasting plasma glucose, FRAP: Ferric Reducing Antioxidant Power, G6P: glucose-6-phosphatase, G6PD: glucose-6-phosphate dehydrogenase, GGT: γ-glutamyl transferase, GPx: Glutathione peroxidase, GR: glutathione-reductase, GSH: Glutathione, GST: Glutathione S-transferase, Hb: hemoglobin, HDL: high-density lipoprotein, HK: hexokinase, HP: hydroperoxides, HOMA-IR: Homeostasis Model Assessment-Insulin Resistance, iNOS: inducible NO synthase, IκB-α: inhibitor of κB, IL-1β: interleukin-1β, IL-6: interleukin-6, IL-10: interleukin-10, iNOS: inducible nitric oxide synthase, i.p.: intraperitoneal, p-IRS-1: phospho-IRS-1, IRS-1: insulin receptor substrate-1; IRS-2: insulin receptor substrate-2; IRβ: insulin receptor beta ITT: insulin tolerance test, LDH: lactate dehydrogenase, LDL: low density lipoprotein, LPL: lipoprotein lipase, LPP: lipid peroxidation products, LPS: lipopolysaccharides, MCV: mean corpuscular volume, MCH: Mean corpuscular hemoglobin, MCHC: mean corpuscular hemoglobin concentration., MCP-1: monocyte chemoattractant protein-1, MDA: malondialdehyde, MDM: monocyte-derived macrophages, β-MHC: beta major histocompatibility complex, MO: Moringa oleifera, MPV: Mean Platelet Volume, NF-κB: nuclear factor-kappa B (NF-κB), NO: nitric oxide, PC: pyruvate carboxylase, PCV: Pack cell volume, PDI: protein disulfide isomerase, P-LCR: Platelet Large Cell Ratio, PDW: Platelet Distribution Width, PGE2: prostaglandin E2

RBC: Red blood cell, RDWa: red blood cell distribution width absolute, ROS: reactive oxygen species, R-QUICKI: revised quantitative insulin sensitivity check index, SCFA: short-chain fatty acid, S.D.: Sprague Dawley, SOD: superoxide dismutase, STZ: streptozotocin, TAC: total antioxidant capacity, TBARS: Thiobarbituric acid-reactive substances, TC: total cholesterol, TG: triglyceride, TNF-α: Tumor Necrosis Factor-Alfa, TP: total protein, TPCC: total protein carbonyl content, WBC: White blood cells

Table S2. Clinical trials of *Moringa oleifera*

**Author, year**	**Type of extracts / main bioactive compounds used**	**Methods of extracts / bioactive compounds preparation**	**Yield of preparation per raw material (wt/wt)**	**Sample size / subjects**	**Treatments and control**	**Duration**	**Effects**	**Adverse events (AE)**
Anthanont, 2016 [103]	Leaf powder capsules	Dried MO leaves were powdered and filled into a gelatin capsule.	Not reported	10 / healthy subjects	Baseline: 0 g,<br>Week 2: 1 g<br>Week 4: 2 g<br>Week 6: 4 g.	8 weeks	- No change in BUN, Cr, AST, and ALT, plasma glucose<br>- ↑ plasma insulin<br>- ↑ insulin secretion.	No AE up to high dose (4 g) administration.
Dominguez-Rodriguez M et al, 2016 [104]	MO. leaves extract	Not reported	Not reported	Crossover study<br>Placebo: 24<br>Treatment: 24 / Obese type 2 diabetics treated with metformin subjects	NA	Period 1: 10 weeks<br>Washout: 2 weeks<br>Period 2: 10 weeks	- ↓ BMI, insulin<br>- Trends to LDL and HbA1c reduction.<br>- ↓ BP.<br>- ↑ HDL	Not reported
Ezzat SM et al., 2020 [41]	MO leaves hard gelatin capsules	Powdered MO leaves were macerated using 70% ethanol and evaporated to yield dried ethanol extract.	10%	15 subjects / who were overweight or obese	400 mg/capsule	8 weeks	- ↓ significantly in BMI, TC, and LDL vs. baseline.<br>- Normalization of AST, ALT, and alanine.	Not reported
Ifeoma, 2020 [105]	Steamed MO	Not reported	Not reported	Control placebo: 6 subjects	Group 1: control<br>Group 2: 20 g	14 days	- No difference in the waist	Not reported

	leaves			Treatment:<br>3 groups, with each group, consists of 6 subjects / T2DM	Group 3: 40 g<br>Group 4: 60 g		circumference, waist-hip ratio, and FBG in all groups.<br>- ↓ SBP, no difference in DBP and TC in Groups 3 and 4.<br>- ↓ LDL in Group 2.<br>- ↑ TG and LDL in Group 3.<br>- ↑ HDL in groups 1, 2, and 4.<br>- ↓ Hb, packed cell volume, WBC, in Group 4. Significant changes observed in the parameters assessed were not dose-dependent.	
Leone A et al., 2018 [106]	Moringa oleifera leaf powder	The leaves were dried through a shade-dried and ground to a fine powder with an electric grinder.	Not reported	10 nondiabetic and 17 diabetic subjects, each received both control and Moringa oleifera leaf powder	20 g added to a meal	Single administration	Lower increment of postprandial blood glucose in diabetic subjects	Not reported

Sandoval & Jimeno, 2013 [107]	MO powdered leaf capsules	Leaves were dried and capsuled	Not reported	Placebo: 35 subjects<br>Treatment group: 33 subjects / High baseline LDL-c patients	Placebo: 2 capsules 3x a day placebo capsule<br>Treatment group: 2 capsules 3x a day (350 mg/capsule or 2100 mg/day)	30 days	A similar reduction of LDL-c vs. placebo	No serious adverse effects
Taweerutchana R et al., 2017 [108]	MO powdered leaf capsules	Dried leaves of MO, were ground, sifted to be powder. The powder was filled into capsule shells.	Not reported	Placebo: 16 subjects<br>Treatment group: 16 subjects / Therapy naïve T2DM patients	Placebo: 8 placebo capsules<br>Treatment group: 4 grams/day (8 capsules)<br>4 capsules each before breakfast and dinner time (500 mg MO powdered leaf/capsules)	4 weeks	no effect on glycemic control	No adverse effects

AE: adverse events, ALT: alanine aminotransferase, AST: aspartate aminotransferase, BUN: Blood urea nitrogen, BP: blood pressure, BMI: body mass index, BW: body weight, Cr: Creatinine, DBP: Diastolic blood pressure, FBG: fasting blood glucose, HDL: high-density lipoprotein, LDL: low-density lipoprotein, TC: total cholesterol, MO: Moringa oleifera, SBP systolic blood pressure, T2DM: type 2 diabetes mellitus TG: triglyceride, WBC: white blood cells.

REFERENCES

[1] S.O. Abarikwu, S. Benjamin, S.G. Ebah, G. Obilor, and G. Agbam, Protective effect of Moringa oleifera oil against HgCl2-induced hepato- and nephrotoxicity in rats. J Basic Clin Physiol Pharmacol 28 (2017) 337-345.

[2] A. Abd El Latif, S. El Bialy Bel, H.D. Mahboub, and M.A. Abd Eldaim, Moringa oleifera leaf extract ameliorates alloxan-induced diabetes in rats by regeneration of β cells and reduction of pyruvate carboxylase expression. Biochem Cell Biol 92 (2014) 413-9.

[3] M.A. Abd Eldaim, A. Shaban Abd Elrasoul, and S.A. Abd Elaziz, An aqueous extract from Moringa oleifera leaves ameliorates hepatotoxicity in alloxan-induced diabetic rats. Biochem Cell Biol 95 (2017) 524-530.

[4] N.Z. Abd Rani, E. Kumolosasi, M. Jasamai, J.A. Jamal, K.W. Lam, and K. Husain, In vitro anti-allergic activity of Moringa oleifera Lam. extracts and their isolated compounds. BMC Complement Altern Med 19 (2019) 361.

[5] Y.M. Abd-Elhakim, W.A. Mohamed, K.M.E. Bohi, H.A. Ali, F.A. Mahmoud, and T.M. Saber, Prevention of melamine-induced hepatorenal impairment by an ethanolic extract of Moringa oleifera: Changes in KIM-1, TIMP-1, oxidative stress, apoptosis, and inflammation-related genes. Gene 764 (2021) 145083.

[6] M.E. Abdel Fattah, H.M. Sobhy, A. Reda, and H.M.A. Abdelrazek, Hepatoprotective effect of Moringa oleifera leaves aquatic extract against lead acetate-induced liver injury in male Wistar rats. Environ Sci Pollut Res Int 27 (2020) 43028-43043.

[7] M.M. Abdel-Daim, S. Alkahtani, R. Almeer, and G. Albasher, Alleviation of lead acetate-induced nephrotoxicity by Moringa oleifera extract in rats: highlighting the antioxidant, anti-inflammatory, and antiapoptotic activities. Environ Sci Pollut Res Int 27 (2020) 33723-33731.

[8] M.M. Abdel-Daim, S.R. Khalil, A. Awad, E.H. Abu Zeid, R.A. El-Aziz, and H.A. El-Serehy, Ethanolic Extract of Moringa oleifera Leaves Influences NF-κB Signaling Pathway to Restore Kidney Tissue from Cobalt-Mediated Oxidative Injury and Inflammation in Rats. Nutrients 12 (2020).

[9] K.H. Abdou, W.A. Moselhy, H.M. Mohamed, E.S. El-Nahass, and A.G. Khalifa, Moringa oleifera Leaves Extract Protects Titanium Dioxide Nanoparticles-Induced Nephrotoxicity via Nrf2/HO-1 Signaling and Amelioration of Oxidative Stress. Biol Trace Elem Res 187 (2019) 181-191.

[10] S.M. Abou-Zeid, A.I. Ahmed, A. Awad, W.A. Mohammed, M.M.M. Metwally, R. Almeer, M.M. Abdel-Daim, and S.R. Khalil, Moringa oleifera ethanolic extract attenuates tilmicosin-induced renal damage in male rats via suppression of oxidative stress, inflammatory injury, and intermediate filament proteins mRNA expression. Biomed Pharmacother 133 (2021) 110997.

[11] E.H. Abu-Zeid, D.M. Abdel Fattah, A.H. Arisha, T.A. Ismail, D.M. Alsadek, M.M.M. Metwally, A.A. El-Sayed, and A.T. Khalil, Protective prospects of eco-friendly synthesized selenium nanoparticles using Moringa oleifera or Moringa oleifera leaf extract against melamine induced nephrotoxicity in male rats. Ecotoxicol Environ Saf 221 (2021) 112424.

[12] A.A. Adedapo, O.O. Falayi, and A.A. Oyagbemi, Evaluation of the analgesic, anti-inflammatory, antioxidant, phytochemical, and toxicological properties of the methanolic

leaf extract of commercially processed Moringa oleifera in some laboratory animals. J Basic Clin Physiol Pharmacol 26 (2015) 491-9.
[13] A.B. Aa, J. Om, E. Ts, and A. Ga, Preliminary phytochemical screening, antioxidant and antihyperglycaemic activity of Moringa oleifera leaf extracts. Pak J Pharm Sci 30 (2017) 2217-2222.
[14] D. Aekthammarat, P. Pannangpetch, and P. Tangsucharit, Moringa oleifera leaf extract lowers high blood pressure by alleviating vascular dysfunction and decreasing oxidative stress in L-NAME hypertensive rats. Phytomedicine 54 (2019) 9-16.
[15] D. Aekthammarat, P. Pannangpetch, and P. Tangsucharit, Moringa oleifera leaf extract induces vasorelaxation via endothelium-dependent hyperpolarization and calcium channel blockade in mesenteric arterial beds isolated from L-NAME hypertensive rats. Clin Exp Hypertens 42 (2020) 490-501.
[16] D. Aekthammarat, P. Tangsucharit, P. Pannangpetch, T. Sriwantana, and N. Sibmooh, Moringa oleifera leaf extract enhances endothelial nitric oxide production leading to relaxation of resistance artery and lowering of arterial blood pressure. Biomed Pharmacother 130 (2020) 110605.
[17] B.Y. Aju, R. Rajalakshmi, and S. Mini, Protective role of Moringa oleifera leaf extract on cardiac antioxidant status and lipid peroxidation in streptozotocin-induced diabetic rats. Heliyon 5 (2019) e02935.
[18] A.S. Akinrinde, O. Oduwole, F.J. Akinrinmade, and F.B. Bolaji-Alabi, Nephroprotective effect of methanol extract of Moringa oleifera leaves on acute kidney injury induced by ischemia-reperfusion in rats. Afr Health Sci 20 (2020) 1382-1396.
[19] A.L. Al-Malki, and H.A. El Rabey, The antidiabetic effect of low doses of Moringa oleifera Lam. seeds on streptozotocin-induced diabetes and diabetic nephropathy in male rats. Biomed Res Int 2015 (2015) 381040.
[20] G. Albasher, S. Al Kahtani, M.S. Alwahibi, and R. Almeer, Effect of Moringa oleifera Lam. methanolic extract on lead-induced oxidative stress-mediated hepatic damage and inflammation in rats. Environ Sci Pollut Res Int 27 (2020) 19877-19887.
[21] F. Alhakmani, S. Kumar, and S.A. Khan, Estimation of total phenolic content, in-vitro antioxidant and anti-inflammatory activity of flowers of Moringa oleifera. Asian Pac J Trop Biomed 3 (2013) 623-7; discussion 626-7.
[22] S. Anudeep, V.K. Prasanna, S.M. Adya, and C. Radha, Characterization of soluble dietary fiber from Moringa oleifera seeds and its immunomodulatory effects. Int J Biol Macromol 91 (2016) 656-62.
[23] L.C. Araújo, J.S. Aguiar, T.H. Napoleão, F.V. Mota, A.L. Barros, M.C. Moura, M.C. Coriolano, L.C. Coelho, T.G. Silva, and P.M. Paiva, Evaluation of cytotoxic and anti-inflammatory activities of extracts and lectins from Moringa oleifera seeds. PLoS One 8 (2013) e81973.
[24] R.O. Arise, O.R. Aburo, S.T. Farohunbi, and A.A. Adewale, Antidiabetic and antioxidant activities of ethanolic extract of dried flowers of Moringa oleifera in streptozotocin-induced diabetic rats. Acta Facultatis Medicae Naissensis 33 (2016) 259-272.
[25] A. Atta, H. Soufy, S. Nasr, A. Soliman, S. Nassar, A.H. Almaweri, T. El-Aziz, H. Desouky3and, and A. Abdalla, Hepatoprotective and antioxidant effects of methanol extract of Moringa oleifera leaves in rats. Wulfenia 24 (2017).

[26] E.S. Attakpa, G.A. Bertin, N.W. Chabi, J.M. Ategbo, B. Seri, and N.A. Khan, Moringa oleifera-rich diet and T cell calcium signaling in spontaneously hypertensive rats. Physiol Res 66 (2017) 753-767.
[27] S.B. Azad, P. Ansari, S. Azam, S.M. Hossain, M.I. Shahid, M. Hasan, and J.M.A. Hannan, Anti-hyperglycaemic activity of Moringa oleifera is partly mediated by carbohydrase inhibition and glucose-fiber binding. Biosci Rep 37 (2017).
[28] M. Azevedo Í, I. Araújo-Filho, M.M.A. Teixeira, M. Moreira, and A.C. Medeiros, Wound healing of diabetic rats treated with Moringa oleifera extract. Acta Cir Bras 33 (2018) 799-805.
[29] Y. Bao, J. Xiao, Z. Weng, X. Lu, X. Shen, and F. Wang, A phenolic glycoside from Moringa oleifera Lam. improves the carbohydrate and lipid metabolisms through AMPK in db/db mice. Food Chem 311 (2020) 125948.
[30] N. Bitrus, I. Uchendu, and O. Orji, The role of crude methanol leaf extract of moringa oleifera in protection against hyperlipidemia and cardiomyopathy in albino rat fed a high cholesterol diet and carbimazole. Pharmacologyonline 3 (2018) 261-271.
[31] K.H. Chen, Y.J. Chen, C.H. Yang, K.W. Liu, J.L. Chang, S.F. Pan, T.B. Lin, and M.J. Chen, Attenuation of the extract from Moringa oleifera on monocrotaline-induced pulmonary hypertension in rats. Chin J Physiol 55 (2012) 22-30.
[32] M. Cheraghi, M. Namdari, H. Daraee, and B. Negahdari, Cardioprotective effect of magnetic hydrogel nanocomposite loaded N,α-L-rhamnopyranosyl vincosamide isolated from Moringa oleifera leaves against doxorubicin-induced cardiac toxicity in rats: in vitro and in vivo studies. J Microencapsul 34 (2017) 335-341.
[33] C.Y. Chin, P.Y. Ng, and S.F. Ng, Moringa oleifera standardized aqueous leaf extract-loaded hydrocolloid film dressing: in vivo dermal safety and wound healing evaluation in STZ/HFD diabetic rat model. Drug Deliv Transl Res 9 (2019) 453-468.
[34] P. Chumark, P. Khunawat, Y. Sanvarinda, S. Phornchirasilp, N.P. Morales, L. Phivthong-Ngam, P. Ratanachamnong, S. Srisawat, and K.U. Pongrapeeporn, The in vitro and ex vivo antioxidant properties, hypolipidaemic and antiatherosclerotic activities of water extract of Moringa oleifera Lam. leaves. J Ethnopharmacol 116 (2008) 439-46.
[35] C. Cui, S. Chen, X. Wang, G. Yuan, F. Jiang, X. Chen, and L. Wang, Characterization of Moringa oleifera roots polysaccharide MRP-1 with anti-inflammatory effect. Int J Biol Macromol 132 (2019) 844-851.
[36] N. Das, K. Sikder, S. Ghosh, B. Fromenty, and S. Dey, Moringa oleifera Lam. leaf extract prevents early liver injury and restores antioxidant status in mice fed with a high-fat diet. Indian J Exp Biol 50 (2012) 404-12.
[37] A.R. Yurre, J. Silva, M. Torres, E.L. Martins, I.P. Ramos, W. Silva, J.D.S. Sarpa, C. Guedes, T.H. Napoleão, L. Coelho, P.M.G. Paiva, and E. Medei, Evaluation of the Cardiac Effects of a Water-Soluble Lectin (Wsmol) from Moringa Oleifera Seeds. Arq Bras Cardiol 114 (2020) 1029-1037.
[38] Z. Dou, C. Chen, and X. Fu, Bioaccessibility, antioxidant activity and modulation effect on gut microbiota of bioactive compounds from Moringa oleifera Lam. leaves during digestion and fermentation in vitro. Food Funct 10 (2019) 5070-5079.
[39] C.O. Edeogu, M.E. Kalu, A.C. Famurewa, N.T. Asogwa, G.N. Onyeji, and K.O. Ikpemo, Nephroprotective Effect of Moringa Oleifera Seed Oil on Gentamicin-Induced Nephrotoxicity in Rats: Biochemical Evaluation of Antioxidant, Anti-inflammatory, and Antiapoptotic Pathways. J Am Coll Nutr 39 (2020) 307-315.

[40] H. El Rabey, J. Khan, F.M. Almutairi, and M. Elbakry, The low dose of drumsticks (Moringa oleifera L.) seed powder ameliorates blood cholesterol in the hypercholesterolemic male rat. Indian Journal of Biochemistry and Biophysics 54 (2017) 306-313.
[41] S.M. Ezzat, M.H. El Bishbishy, N.M. Aborehab, M.M. Salama, A. Hasheesh, A.A. Motaal, H. Rashad, and F.M. Metwally, Upregulation of MC4R and PPAR-α expression mediates the anti-obesity activity of Moringa oleifera Lam. in high-fat diet-induced obesity in rats. J Ethnopharmacol 251 (2020) 112541.
[42] J.W. Fahey, K.L. Wade, K.K. Stephenson, Y. Shi, H. Liu, A.A. Panjwani, C.R. Warrick, and M.E. Olson, A Strategy to Deliver Precise Oral Doses of the Glucosinolates or Isothiocyanates from Moringa oleifera Leaves for Use in Clinical Studies. Nutrients 11 (2019).
[43] X. Gao, Q. Xie, L. Liu, P. Kong, J. Sheng, and H. Xiang, Metabolic adaptation to the aqueous leaf extract of Moringa oleifera Lam.-supplemented diet is related to the modulation of gut microbiota in mice. Appl Microbiol Biotechnol 101 (2017) 5115-5130.
[44] S. Ghasi, E. Nwobodo, and J.O. Ofili, Hypocholesterolemic effects of crude extract of leaf of Moringa oleifera Lam in high-fat diet-fed Wistar rats. J Ethnopharmacol 69 (2000) 21-5.
[45] A.S. Gouda, N.A. El-Nabarawy and S.F. Ibrahim, Moringa oleifera extract (Lam) attenuate Aluminium phosphide-induced acute cardiac toxicity in rats. Toxicol Rep 5 (2018) 209-212.
[46] R. Gupta, M. Mathur, V.K. Bajaj, P. Katariya, S. Yadav, R. Kamal, and R.S. Gupta, Evaluation of antidiabetic and antioxidant activity of Moringa oleifera in experimental diabetes. J Diabetes 4 (2012) 164-71.
[47] L. Huang, C. Yuan, and Y. Wang, Bioactivity-guided Identification of Anti-Adipogenic Isothiocyanates in the Moringa (Moringa oleifera) Seed and Investigation of the Structure-Activity Relationship. Molecules 25 (2020).
[48] Q. Huang, R. Liu, J. Liu, Q. Huang, S. Liu, and Y. Jiang, Integrated Network Pharmacology Analysis and Experimental Validation to Reveal the Mechanism of Anti-Insulin Resistance Effects of Moringa oleifera Seeds. Drug Des Devel Ther 14 (2020) 4069-4084.
[49] H. Irfan, M. Abdullah, N. Khan, and A. Sadikun, Effect of Ethanolic Extract of Moringa oleifera Lam. Leaves on Body Weight and Hyperglycemia of Diabetic Rats. Pakistan Journal of Nutrition 15 (2016) 112-117.
[50] D. Jaiswal, P.K. Rai, A. Kumar, S. Mehta, and G. Watal, Effect of Moringa oleifera Lam. leaves aqueous extract therapy on hyperglycemic rats. J Ethnopharmacol 3(2009) 392 - 6.
[51] D. Jaiswal, P.K. Rai, S. Mehta, S. Chatterji, S. Shukla, D.K. Rai, G. Sharma, B. Sharma, S. Khair, and G. Watal, Role of Moringa oleifera in the regulation of diabetes-induced oxidative stress. Asian Pac J Trop Med 6 (2013) 426-32
[52] A. Jaja-Chimedza, B.L. Graf, C. Simmler, Y. Kim, P. Kuhn, G.F. Pauli, and I. Raskin, Biochemical characterization and anti-inflammatory properties of an isothiocyanate-enriched moringa (Moringa oleifera) seed extract. PLoS One 12 (2017) e0182658.
[53] H. Joung, B. Kim, H. Park, K. Lee, H.-H. Kim, H.-C. Sim, H.-J. Do, C.-K. Hyun, and M.-S. Do, Fermented Moringa oleifera decreases hepatic adiposity and ameliorates glucose intolerance in high-fat diet-induced obese mice. Journal of medicinal food 20 (2017) 439-447.
[54] S.R. Khalil, S.M. Abdel-Motal, M. Abd-Elsalam, N.E. Abd El-Hameed, and A. Awad, Restoring strategy of ethanolic extract of Moringa oleifera leaves against Tilmicosin-

induced cardiac injury in rats: Targeting cell apoptosis-mediated pathways. Gene 730 (2020) 144272.

[55] S. Kumar Gupta, B. Kumar, B.P. Srinivasan, T.C. Nag, S. Srivastava, R. Saxena, and A. Aggarwal, Retinoprotective effects of Moringa oleifera via antioxidant, anti-inflammatory, and anti-angiogenic mechanisms in streptozotocin-induced diabetic rats. J Ocul Pharmacol Ther 29 (2013) 419-26.

[56] C. Li, Z. Dong, B. Zhang, Q. Huang, G. Liu, and X. Fu, Structural characterization and immune enhancement activity of a novel polysaccharide from Moringa oleifera leaves. Carbohydr Polym 234 (2020) 115897.

[57] Y.J. Li, Q.Q. Ji, Z. Wang, L.H. Shen, and B. He, Moringa oleifera seeds mitigate myocardial injury and prevent ventricular failure induced by myocardial infarction. Am J Transl Res 12 (2020) 4511-4521.

[58] P.C. Liao, M.H. Lai, K.P. Hsu, Y.H. Kuo, J. Chen, M.C. Tsai, C.X. Li, X.J. Yin, N. Jeyashoke, and L.K. Chao, Identification of β-Sitosterol as in Vitro Anti-Inflammatory Constituent in Moringa oleifera. J Agric Food Chem 66 (2018) 10748-10759.

[59] M. López, M. Ríos-Silva, M. Huerta, Y. Cárdenas, J.A. Bricio-Barrios, M.I. Díaz-Reval, Z. Urzúa, M. Huerta-Trujillo, K. López-Quezada, and X. Trujillo, Effects of Moringa oleifera leaf powder on metabolic syndrome induced in male Wistar rats: a preliminary study. J Int Med Res 46 (2018) 3327-3336.

[60] T. Luetragoon, R. Pankla Sranujit, C. Noysang, Y. Thongsri, P. Potup, N. Suphrom, N. Nuengchamnong, and K. Usuwanthim, Bioactive Compounds in Moringa oleifera Lam. Leaves Inhibit the Pro-Inflammatory Mediators in Lipopolysaccharide-Induced Human Monocyte-derived Macrophages. Molecules 25 (2020).

[61] L. Mabrouki, I. Rjeibi, J. Taleb, and L. Zourgui, Cardiac Ameliorative Effect of Moringa oleifera Leaf Extract in High-Fat Diet-Induced Obesity in Rat Model. Biomed Res Int 2020 (2020) 6583603.

[62] H. Madkhali, M. Alharthy, M. Asiri, M. Ganaie, M. Ansari, N.U. Rehman and A. Hamad, Moringa oleifera Lam. (family Moringaceae) leaf extract attenuate high-fat diet-induced dyslipidemia and vascular endothelium dysfunction in Wistar albino rats. 18 (2019) 2597-2604.

[63] M. Mapfumo, B.W. Lembede, A.R. Ndhlala, and E. Chivandi, Effect of crude Moringa oleifera Lam. seed extract on the blood markers of metabolic syndrome in high-fructose diet-fed growing Sprague-Dawley rats. J Complement Integr Med 17 (2019).

[64] K. Mehta, R. Balaraman, A.H. Amin, P.A. Bafna, and O.D. Gulati, Effect of fruits of Moringa oleifera on the lipid profile of normal and hypercholesterolaemic rabbits. J Ethnopharmacol 86 (2003) 191-5.

[65] A.A. Muhammad, P. Arulselvan, P.S. Cheah, F. Abas, and S. Fakurazi, Evaluation of wound healing properties of bioactive aqueous fraction from Moringa oleifera Lam on an experimentally induced diabetic animal model. Drug Des Devel Ther 10 (2016) 1715-30.

[66] G. Murillo, M. Almatrafi, M. Vergara-Jimenez, and M.L. Fernandez, Moringa leaf powder modulate reverse cholesterol transport without changing plasma lipids in guinea. The FASEB Journal 31 (2017) 647.2-647.2.

[67] A.O. Nafiu, R.O. Akomolafe, Q.K. Alabi, C.O. Idowu, and O.O. Odujoko, Effect of fatty acids from ethanol extract of Moringa oleifera seeds on kidney function impairment and oxidative stress induced by gentamicin in rats. Biomed Pharmacother 117 (2019) 109154.

[68] M. Nandave, S.K. Ojha, S. Joshi, S. Kumari, and D.S. Arya, Moringa oleifera leaf extract prevents isoproterenol-induced myocardial damage in rats: evidence for an antioxidant, antiperoxidative, and cardioprotective intervention. J Med Food 12 (2009) 47-55.

[69] M. Ndong, M. Uehara, S. Katsumata, S. Sato, and K. Suzuki, Preventive effects of Moringa oleifera (Lam) on hyperlipidemia and hepatocyte ultrastructural changes in iron-deficient rats. Biosci Biotechnol Biochem 71 (2007) 1826-33.

[70] O. Ofem, E. Ani, A. Archibong, and J. Ufford, Variations in blood parameters of high salt loaded rats following administration of Moringa oleifera leaf extract. Trends Med Res 10 (2015) 97-105.

[71] L.A. Olayaki, J.E. Irekpita, M.T. Yakubu, and O.O. Ojo, Methanolic extract of Moringa oleifera leaves improves glucose tolerance, glycogen synthesis, and lipid metabolism in alloxan-induced diabetic rats. J Basic Clin Physiol Pharmacol 26 (2015) 585-93.

[72] T.L.C. Oldoni, N. Merlin, T.C. Bicas, A. Prasniewski, S.T. Carpes, J. Ascari, S.M. de Alencar, A.P. Massarioli, M.D. Bagatini, R. Morales, and G. Thomé, Antihyperglycemic activity of crude extract and isolation of phenolic compounds with antioxidant activity from Moringa oleifera Lam. leaves grown in Southern Brazil. Food Res Int 141 (2021) 110082.

[73] E.I. Omodanisi, Y.G. Aboua, and O.O. Oguntibeju, Assessment of the Anti-Hyperglycaemic, Anti-Inflammatory and Antioxidant Activities of the Methanol Extract of Moringa Oleifera in Diabetes-Induced Nephrotoxic Male Wistar Rats. Molecules 22 (2017).

[74] M. Ouédraogo, A. Lamien-Sanou, N. Ramdé, A.S. Ouédraogo, M. Ouédraogo, S.P. Zongo, O. Goumbri, P. Duez, and P.I. Guissou, Protective effect of Moringa oleifera leaves against gentamicin-induced nephrotoxicity in rabbits. Experimental and Toxicologic Pathology 65 (2013) 335-339.

[75] S. Panda, A. Kar, P. Sharma, and A. Sharma, Cardioprotective potential of N, α-l-rhamnopyranosyl vincosamide, an indole alkaloid, isolated from the leaves of Moringa oleifera in isoproterenol-induced cardiotoxic rats: In vivo and in vitro studies. Bioorganic & medicinal chemistry letters 23 (2013) 959-962.

[76] S. Panda, Butanolic fraction of Moringa oleifera Lam. (Moringaceae) attenuates isoproterenol-induced cardiac necrosis and oxidative stress in rats: an EPR study. EXCLI journal 14 (2015) 64.

[77] P.C. Paula, D.O. Sousa, J.T. Oliveira, A.F. Carvalho, B.G. Alves, M.L. Pereira, D.F. Farias, M.P. Viana, F.A. Santos, and T.C. Morais, A protein isolate from Moringa oleifera leaves has hypoglycemic and antioxidant effects in alloxan-induced diabetic mice. Molecules 22 (2017) 271.

[78] M. Rajanandh, M. Satishkumar, K. Elango, and B. Suresh, Moringa oleifera Lam. Herbal medicine for hyperlipidemia: A preclinical report. Asian Pacific Journal of Tropical Disease 2 (2012) S790-S795.

[79] J.I. Randriamboavonjy, G. Loirand, N. Vaillant, B. Lauzier, S. Derbré, S. Michalet, P. Pacaud, and A. Tesse, Cardiac protective effects of Moringa oleifera seeds in spontaneously hypertensive rats. American journal of hypertension 29 (2016) 873-881.

[80] J.I. Randriamboavonjy, M. Rio, P. Pacaud, G. Loirand, and A. Tesse, Moringa oleifera seeds attenuate vascular oxidative and nitrosative stresses in spontaneously hypertensive rats. Oxidative medicine and cellular longevity 2017 (2017).

[81] V. Reddy, A. Urooj, S. Sairam, F. Ahmed, and N. Prasad, Hypocholesterolemic effect of Moringa oleifera polyphenols in rats fed high fat-cholesterol diet. Malaysian Journal of Nutrition 23 (2017) 473-478.

[82] W.A. Saka, T.E. Ayoade, T.M. Akhigbe, and R.E. Akhigbe, Moringa oleifera seed oil partially abrogates 2, 3-dichlorovinyl dimethyl phosphate (Dichlorvos)-induced cardiac injury in rats: evidence for the role of oxidative stress. Journal of Basic and Clinical Physiology and Pharmacology 32 (2021) 237-246.
[83] W. Sangkitikomol, A. Rocejanasaroj, and T. Tencomnao, Effect of Moringa oleifera on advanced glycation end-product formation and lipid metabolism gene expression in HepG2 cells. Genetics and Molecular Research 13 (2014) 723-735.
[84] S. SHAIKH, S. SHAIKH, F. SAYYED, P. KHAN, S. SHAIKH, and S. JAMKHEDKAR, Traditional plants: in vitro analysis of medicinal properties. International Journal of Pharmaceutical Research (2020).
[85] H.N. Sholapur, and B.M. Patil, Effect of Moringa oleifera bark extracts on dexamethasone-induced insulin resistance in rats. Drug Res (Stuttg) 63 (2013) 527-31.
[86] M. Sinha, D.K. Das, S. Bhattacharjee, S. Majumdar, and S. Dey, Leaf extract of Moringa oleifera prevents ionizing radiation-induced oxidative stress in mice. J Med Food 14 (2011) 1167-72.
[87] M.M. Soliman, A. Aldhahrani, A. Alkhedaide, M.A. Nassan, F. Althobaiti, and W.A. Mohamed, The ameliorative impacts of Moringa oleifera leaf extract against oxidative stress and methotrexate-induced hepato-renal dysfunction. Biomedicine & Pharmacotherapy 128 (2020) 110259.
[88] C. Sun, W. Li, Y. Liu, W. Deng, M. Adu-Frimpong, H. Zhang, Q. Wang, J. Yu, and X. Xu, In vitro/in vivo hepatoprotective properties of 1-O-(4-hydroxymethylphenyl)-α-L-rhamnopyranoside from Moringa oleifera seeds against carbon tetrachloride-induced hepatic injury. Food and Chemical Toxicology 131 (2019) 110531.
[89] W.S. Tan, P. Arulselvan, G. Karthivashan, and S. Fakurazi, Moringa oleifera flower extract suppresses the activation of inflammatory mediators in lipopolysaccharide-stimulated RAW 264.7 macrophages via NF-κB pathway. Mediators of inflammation 2015 (2015).
[90] Y. Tang, E.-J. Choi, W.C. Han, M. Oh, J. Kim, J.-Y. Hwang, P.-J. Park, S.-H. Moon, Y.-S. Kim, and E.-K. Kim, Moringa oleifera from Cambodia ameliorates oxidative stress, hyperglycemia, and kidney dysfunction in type 2 diabetic mice. Journal of medicinal food 20 (2017) 502-510.
[91] H. Tian, Y. Liang, G. Liu, Y. Li, M. Deng, D. Liu, Y. Guo, and B. Sun, Moringa oleifera polysaccharides regulates caecal microbiota and small intestinal metabolic profile in C57BL/6 mice. International Journal of Biological Macromolecules 182 (2021) 595-611.
[92] S. Umar, Z. Mohammed, A. Nuhu, K. Musa, and Y. Tanko, Evaluation of Hypoglycaemic and Antioxidant Activity of Moringa oleifera Root in Normal and Alloxan-Induced Diabetic Rats. Trop J Nat Prod Res 2 (2018) 401-408.
[93] O.W. Vargas-Tineo, D.M. Segura-Muñoz, L.K. Becerra-Gutiérrez, J.P. Amado-Tineo, and H. Silva-Díaz, Efecto hipoglicemiante de Moringa oleifera (moringa) comparado con smallanthus sonchifolius (yacón) en Rattus norvegicus con diabetes mellitus inducida. Revista Peruana de Medicina Experimental y Salud Pública 37 (2020) 478-484.
[94] M.K. Velaga, L.K. Daughtry, A.C. Jones, P.R. Yallapragada, S. Rajanna, and B. Rajanna, Attenuation of lead-induced oxidative stress in rat brain, liver, kidney, and blood of male Wistar rats by Moringa oleifera seed powder. Journal of Environmental Pathology, Toxicology and Oncology 33 (2014).
[95] N. Vera-Nunez, A.R. Guirao, J.D.F. SILVA, I.P. Ramos, M.K. Torres, L.C.B. Coelho, T.H. Napoleão, P. PAIVA, G. MARIA, and E. MEDEI, Water-soluble lectin (WSMoL) from

Moringa oleifera seeds treatment recovers glycemic levels and improves left ventricular ejection fraction on Type-2 Diabetes mice model. Anais da Academia Brasileira de Ciências 93 (2021).
[96] A. Villarruel-López, D. López-de la Mora, O. Vázquez-Paulino, A. Puebla-Mora, M.R. Torres-Vitela, L. Guerrero-Quiroz, and K. Nuño, Effect of Moringa oleifera consumption on diabetic rats. BMC complementary and alternative medicine 18 (2018) 1-10.
[97] F. Wang, Y. Bao, X. Shen, G. Zengin, Y. Lyu, J. Xiao, and Z. Weng, Niazirin from Moringa oleifera Lam. attenuates high glucose-induced oxidative stress through PKCζ/Nox4 pathway. Phytomedicine (2019) 153066.
[98] F. Wang, Y.-F. Bao, J.-J. Si, Y. Duan, Z.-B. Weng, and X.-C. Shen, The beneficial effects of a polysaccharide from Moringa oleifera leaf on gut microecology in mice. Journal of medicinal food 22 (2019) 907-918.
[99] F. Wang, H.-H. Zhong, W.-K. Chen, Q.-P. Liu, C.-Y. Li, Y.-F. Zheng, and G.-P. Peng, Potential hypoglycaemic activity phenolic glycosides from Moringa oleifera seeds. Natural product research 31 (2017) 1869-1874.
[100] C. Waterman, P. Rojas-Silva, T.B. Tumer, P. Kuhn, A.J. Richard, S. Wicks, J.M. Stephens, Z. Wang, R. Mynatt, and W. Cefalu, Isothiocyanate-rich Moringa oleifera extract reduces weight gain, insulin resistance, and hepatic gluconeogenesis in mice. Molecular nutrition & food research 59 (2015) 1013-1024.
[101] Y. Yang, C.-Y. Shi, J. Xie, J.-H. Dai, S.-L. He, and Y. Tian, Identification of potential dipeptidyl peptidase (DPP)-IV inhibitors among Moringa oleifera phytochemicals by virtual screening, molecular docking analysis, ADME/T-based prediction, and in vitro analyses. Molecules 25 (2020) 189.
[102] H.D. Yassa and A.F. Tohamy, Extract of Moringa oleifera leaves ameliorates streptozotocin-induced Diabetes mellitus in adult rats. Acta Histochemica 116 (2014) 844-854.
[103] P. Anthanont, N. Lumlerdkij, P. Akarasereenont, S. Vannasaeng, and A. Sriwijitkamol, Moringa Oleifera Leaf Increases Insulin Secretion after Single Dose Administration: A Preliminary Study in Healthy Subjects. J Med Assoc Thai 99 (2016) 308-13.
[104] M. Dominguez-Rodriguez, N.G. Valenzuela-Rubio, D.A. Ochoa-Acosta, J.A. Fierros-Valdez, F.H. Castro-Sanchez, and M. Vergara-Jimenez, The Effect of Moringa Oleífera Leaves in Anthropometric and Biochemical Parameters in Obese Type 2 Diabetes Mellitus Subjects. The FASEB Journal 30 (2016) 1176.21-1176.21.
[105] C.A. Ifeoma, Effect of moringa oleifera leaves on the blood glucose, blood pressure, lipid profile and hematological parameters of type 2 diabetics in a rural Nigerian community., South African Medical Research Council, 2020, pp. PACTR202005626842621.
[106] A. Leone, S. Bertoli, S. Di Lello, A. Bassoli, S. Ravasenghi, G. Borgonovo, F. Forlani, and A. Battezzati, Effect of Moringa oleifera Leaf Powder on Postprandial Blood Glucose Response: In Vivo Study on Saharawi People Living in Refugee Camps. Nutrients 10 (2018).
[107] M.A.S. Sandoval, and C.A. Jimeno, Effect of malunggay (Moringa oleifera) capsules on lipid and glucose levels. Acta Medica Philippina 47 (2013).
[108] R. Taweerutchana, N. Lumlerdkij, S. Vannasaeng, P. Akarasereenont, and A. Sriwijitkamol, Effect of Moringa oleifera Leaf Capsules on Glycemic Control in Therapy-Naïve Type 2 Diabetes Patients: A Randomized Placebo-Controlled Study. Evid Based Complement Alternat Med 2017 (2017) 6581390-6581390.
